# Potential *α*-Glucosidase Inhibitors from the Deep-Sea Sediment-Derived Fungus *Aspergillus insulicola*

**DOI:** 10.3390/md21030157

**Published:** 2023-02-26

**Authors:** Weibo Zhao, Yanbo Zeng, Wenjun Chang, Huiqin Chen, Hao Wang, Haofu Dai, Fang Lv

**Affiliations:** 1 Beijing Key Laboratory for Separation and Analysis in Biomedicine and Pharmaceuticals, School of Life Science, Beijing Institute of Technology, Beijing 100081, China; 2 Hainan Provincial Key Laboratory for Functional Components Research and Utilization of Marine Bio-Resources, Institute of Tropical Bioscience and Biotechnology, Chinese Academy of Tropical Agricultural Sciences & Key Laboratory for Biology and Genetic Resources of Tropical Crops of Hainan Province, Hainan Institute for Tropical Agricultural Resources, Haikou 571101, China; 3 Zhanjiang Experimental Station of Chinese Academy of Tropical Agricultural Sciences, Zhanjiang 524013, China

**Keywords:** marine fungus, *Aspergillus insulicola*, phenolic compounds, ECD calculations, *α*-glucosidase inhibition

## Abstract

Three new phenolic compounds, epicocconigrones C–D (**1**–**2**) and flavimycin C (**3**), together with six known phenolic compounds: epicocconigrone A (**4**); 2-(10-formyl-11,13-dihydroxy-12-methoxy-14-methyl)-6,7-dihydroxy-5-methyl-4-benzofurancarboxaldehyde (**5**); epicoccolide B (**6**); eleganketal A (**7**); 1,3-dihydro-5-methoxy-7-methylisobenzofuran (**8**); and 2,3,4-trihydroxy-6-(hydroxymethyl)-5-methylbenzyl-alcohol (**9**), were isolated from fermentation cultures of a deep-sea sediment-derived fungus, *Aspergillus insulicola*. Their planar structures were elucidated based on the 1D and 2D NMR spectra and HRESIMS data. The absolute configurations of compounds **1**–**3** were determined by ECD calculations. Compound **3** represented a rare fully symmetrical isobenzofuran dimer. All compounds were evaluated for their *α*-glucosidase inhibitory activity, and compounds **1**, **4**–**7**, and **9** exhibited more potent *α*-glucosidase inhibitory effect with IC_50_ values ranging from 17.04 to 292.47 μM than positive control acarbose with IC_50_ value of 822.97 μM, indicating that these phenolic compounds could be promising lead compounds of new hypoglycemic drugs.

## 1. Introduction

According to the International Diabetes Federation, 537 million people worldwide were diagnosed with diabetes mellitus in 2021, and about 90 percent of them were type 2 diabetes mellitus (T2DM) [1,2]. T2DM is a chronic metabolic disease that is characterized by postprandial hyperglycemia in the case of insulin resistance and relative lack of insulin [3]. The inhibition of *α*-glucosidase can reduce the cleavage of glucose from disaccharides or oligosaccharides to inhibit postprandial hyperglycemia [4]. Therefore, *α*-glucosidase is a common therapeutic target for the treatment of T2DM [5]. Currently available *α*-glucosidase inhibitors, such as acarbose, voglibose and miglitol, have been used to treat T2DM patients. Nevertheless, the use of these drugs has been associated with serious side effects, such as abdominal distension and diarrhea [6,7]. For this reason, the search for natural, efficient and non-toxic *α*-glucosidase inhibitors provides an attractive strategy for the development of new hypoglycemic drugs.

**Figure 1:**
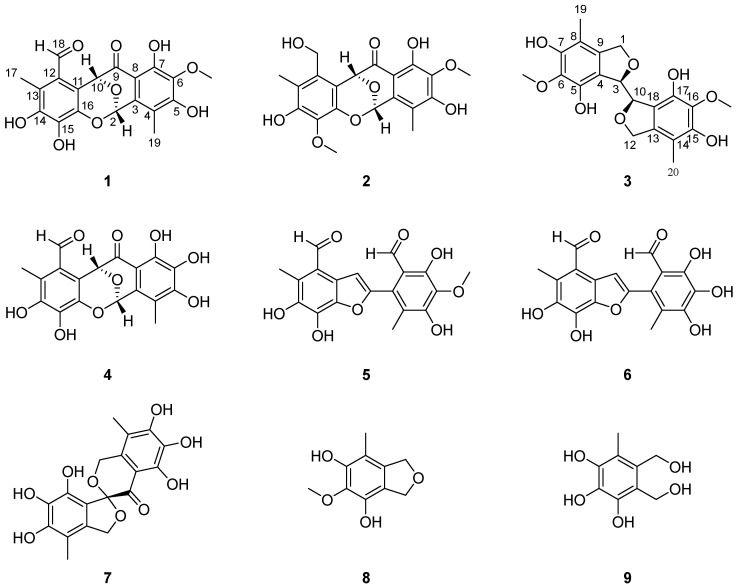
Structures of compounds **1**–**9** from *Aspergillus insulicola*: epicocconigrones C–D (**1**–**2**); flavimycin C (**3**); epicocconigrone A (**4**); 2-(10-formyl-11,13-dihydroxy-12-methoxy-14-methyl)-6,7-dihydroxy-5-methyl-4-benzofurancarboxaldehyde (**5**); epicoccolide B (**6**); eleganketal A (**7**); 1,3-dihydro-5-methoxy-7-methylisobenzofuran (**8**); and 2,3,4-trihydroxy-6-(hydroxymethyl)-5-methylbenzyl-alcohol (**9**).

Phenolic compounds have been proved to be effective *α*-glucosidase inhibitors [8,9,10,11]. Marine phenolic compounds are far less researched than those from terrestrial sources, which could suggest great potential in the ocean to develop novel diabetes drugs [12]. Some marine phenolic compounds isolated from seaweed [13,14] and seagrass [15] have been confirmed to have wonderful *α*-glucosidase inhibitory activity. In order to find more marine phenolic compounds with *α*-glucosidase inhibitory activity, our team studied marine fungi from the South China Sea. *Aspergillus insulicola*, a fungi previously not extensively studied, had great development and utilization value. Previous chemical studies of *A*. *insulicola* have discovered many peptides [16,17,18] and nitrobenzoyl sesquiterpenoids [19,20], which showed significant biological activities, including anti-bacteria [16] and cytotoxic [19,20]. During our ongoing research in finding new compounds with potential bioactivities [21,22,23], a chemical investigation of the deep-sea sediment-derived fungus *A*. *insulicola* led to the isolation and identification of three new phenolic compounds, epicocconigrones C–D (**1**–**2**) and flavimycin C (**3**), together with six known phenolic compounds (**4**–**9**) (Figure 1). All compounds were investigated for their *α*-glucosidase inhibitory activity. Herein, we describe the structure elucidation of the new metabolites as well as the *α*-glucosidase inhibitory activity of the isolated compounds.

## 2. Results and Discussion

### 2.1. Structure Elucidation of New Compounds ***1**–**3***

**Figure 2:**
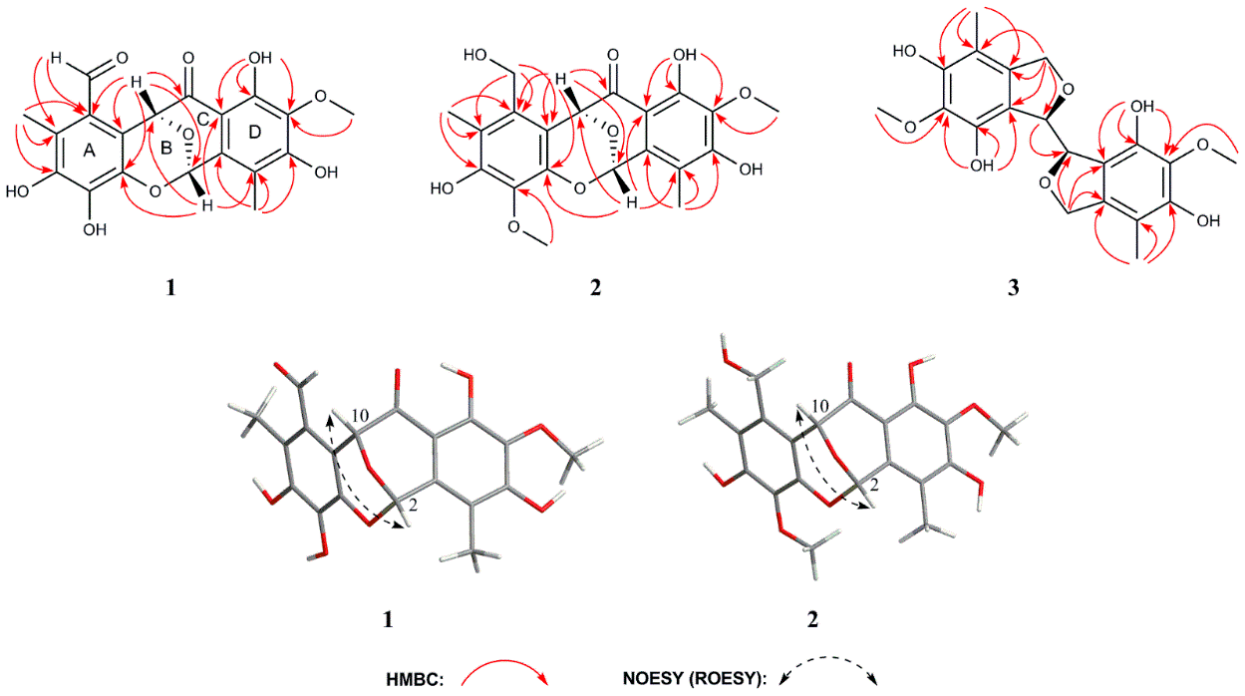
Key HMBC correlations of compounds **1**–**3** and key NOESY/ROESY correlations of compounds **1**–**2**.

**Figure 3:**
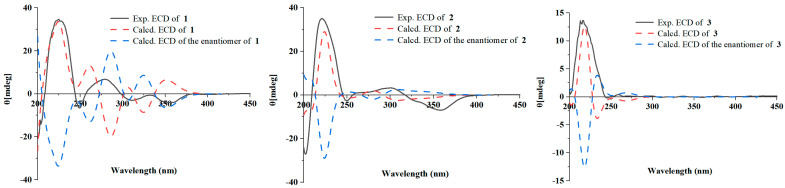
Experimental and calculated ECD spectra of compounds **1**–**3**.

Epicocconigrone C (**1**) was isolated as a yellow solid, and its molecular formula was determined to be C_19_H_16_O_9_ with 12 degrees of unsaturation by HRESIMS data at *m/z* 411.0698 (calcd. 411.0687 for C_19_H_16_O_9_Na, [M + Na]^+^), which was supported by the ^13^C NMR and DEPT spectral data. The IR spectrum of **1** featured typical absorption bands for hydroxyl (3413 cm^−1^) and conjugated ketone (1670 cm^−1^). The ^1^H NMR spectrum (Table 1) of **1** revealed two methyls (*δ*_H_ 2.26 and *δ*_H_ 2.31), one methoxy (*δ*_H_ 3.70), two oxymethines (*δ*_H_ 6.38 and *δ*_H_ 6.83), one aldehyde proton (*δ*_H_ 10.34), and one hydroxyl proton (*δ*_H_ 11.33). The ^13^C NMR (Table 2) and DEPT spectra showed 19 well-resolved carbon atom signals, including one ketone carbonyl (*δ*_C_ 196.9), one aldehydic carbonyl (*δ*_C_ 191.2), two oxygenated tertiary carbons (*δ*_C_ 89.8 and *δ*_C_ 68.6), one methoxy carbon (*δ*_C_ 60.2), two methyls (*δ*_C_ 11.8 and *δ*_C_ 10.2), and twelve olefinic quaternary carbons at *δ*_C_ 156.9−104.5, accounting for 8 degrees of unsaturation. Thus, compound **1** was thought to possess a tetracyclic skeleton. The strong heteronuclear multiple-bond correlation (HMBC) correlations from H-17 (*δ*_H_ 2.31) to C-12 (*δ*_C_ 121.7), C-13 (*δ*_C_ 121.9), and C-14 (*δ*_C_ 144.3), from H-18 (*δ*_H_ 10.34) to C-11 (*δ*_C_ 112.6), C-12, C-13, and C-14, as well as the weak signals from H-17 to C-11, C-15 (*δ*_C_ 138.4), and C-16 (*δ*_C_ 135.8) confirmed the existence of ring A (Figure 2). The HMBC correlations from H-19 (*δ*_H_ 2.26) to C-3 (*δ*_C_ 130.8), C-4 (*δ*_C_ 115.8), and C-5 (*δ*_C_ 156.9), as well as the HMBC correlations from 7-OH (*δ*_H_ 11.33) to C-6, C-7 (*δ*_C_ 153.6), and C-8 (*δ*_C_ 104.5) established the substitution of the aromatic ring D. Furthermore, the HMBC correlations from H-2 (*δ*_H_ 6.83) to C-10 (*δ*_C_ 68.6) and C-16, from H-10 (*δ*_H_ 6.38) to C-2 (*δ*_C_ 89.8), C-11, C-12 and C-16 suggested the presence of two oxygen bridges between C-16/C-2 and C-2/C-10 in ring B, which could be confirmed by the low field chemical shift signal of CH-2 (*δ*_C_ 89.8, *δ*_H_ 6.83). Ring C was established by the HMBC correlations from H-2 to C-4 and C-8, and from H-10 to C-8 and C-9 (*δ*_C_ 196.9). The comprehensive NMR analysis indicated that **1** shared the same oxygen-bridged skeleton with epicocconigrone A (**4**) [24], with the exception that the appearance of 6-OCH_3_ in **1** replaced 6-OH in **4,** which was supported by the HMBC correlation from 6-OCH_3_ (*δ*_H_ 3.70) to C-6 (*δ*_C_ 134.7). Thus, the planar structure of **1** was elucidated as shown (Figure 1), named epicocconigrone C. In the nuclear Overhauser effect spectroscopy (NOESY) spectrum of **1**, the correlation between H-2 and H-10 was indicative of their *cis* relationship (Figure 2). The absolute configuration of **1** was confirmed by the ECD calculation. Its experimental ECD curve for the absolute configurations of 2*S* and 10*R* was consistent with the calculated ECD curve of (2*S*, 10*R*) (Figure 3).

Epicocconigrone D (**2**) was obtained as a yellow solid. The molecular formula of **2** was determined as C_20_H_20_O_9_ with 11 unsaturated degrees by HRESIMS data at *m/z* 427.1004 (calcd. 427.1000 for C_20_H_20_O_9_Na, [M + Na]^+^), which was supported by the ^13^C NMR and DEPT spectral data. The IR spectrum of **2** featured typical absorption bands for hydroxyl (3446 cm^−1^) and conjugated ketone (1626 cm^−1^). The ^1^H NMR spectrum (Table 1) of **2** indicated two methyl groups (*δ*_H_ 2.09 and *δ*_H_ 2.18), two methoxy groups (*δ*_H_ 3.57 and *δ*_H_ 3.69), one methylene (*δ*_H_ 4.32, d, *J* = 12.1 Hz; 4.81 d, *J* = 12.1 Hz), two oxymethines (*δ*_H_ 5.65 and *δ*_H_ 6.76), and two hydroxyl protons (*δ*_H_ 8.92 and *δ*_H_ 11.46). The ^13^C NMR (Table 2) and DEPT spectra revealed 20 carbon atom signals, including one ketone carbonyl (*δ*_C_ 196.8), two oxygenated tertiary carbons (*δ*_C_ 89.9 and *δ*_C_ 70.3), two methoxy carbons (*δ*_C_ 60.3 and *δ*_C_ 60.0), one methylene (*δ*_C_ 55.8), two methyls (*δ*_C_ 11.0 and *δ*_C_ 10.3), and twelve olefinic quaternary carbons. Detailed analysis of 2D NMR spectra of **2** revealed that it had a similar structure to **1**. The major differences in **2** were a hydroxymethylene group and a methoxy group substituted at C-12 and C-15, instead of the aldehyde group and the hydroxyl group, respectively, when compared to **1** (Figure 2), which were further confirmed by the HMBC correlations from H_2_-18 (*δ*_H_ 4.32, 4.81) to C-11 (*δ*_C_ 108.3), C-12 (*δ*_C_ 132.1), and C-13 (*δ*_C_ 118.1), and from 15-OCH_3_ (*δ*_H_ 3.69) to C-15 (*δ*_C_ 134.6). Thus, the planar structure of **2** was elucidated as shown (Figure 1), named epicocconigrone D. The ROESY correlation between H-2 and H-10 indicated their *cis* orientation (Figure 2). The absolute configuration of **2** was understood to be 2*S*, 10*R* by comparing the experimental and simulated ECD curves (Figure 3).

Flavimycin C (**3**) was isolated as a white solid. It had a molecular formula of C_20_H_22_O_8_ with 10 degrees of unsaturation, as determined by HRESIMS data at *m/z* 391.1385 (calcd. 391.1387 for C_20_H_23_O_8_, [M + H]^+^), which was supported by the ^13^C NMR and DEPT spectral data. The IR spectrum of **3** featured typical absorption bands for hydroxyl (3449 cm^−1^). The ^1^H NMR spectrum (Table 1) of **3** exhibited one methyl (*δ*_H_ 1.88), one methoxy (*δ*_H_ 3.64), one methine (*δ*_H_ 4.30), one methylene (*δ*_H_ 4.54 d, *J* = 15.0 Hz; 4.65 d, *J* = 15.0 Hz), and two hydroxyl protons (*δ*_H_ 8.55, *δ*_H_ 8.68). The ^13^C NMR (Table 2) and DEPT spectra displayed 10 well-resolved carbon atom signals, dividing into six quaternary carbons that were assigned to one benzene ring, one methylene (*δ*_C_ 65.8), one methine (*δ*_C_ 66.1), one methoxy carbon (*δ*_C_ 60.2), and one methyl (*δ*_C_ 9.5). The NMR data of **3** were very similar to those of **8** except for the absence of the methylene signal, and instead, the presence of the methine signal of C-3 (*δ*_H_ 4.30/*δ*_C_ 66.1) in **3**. Combined with molecular formula, **3** was deduced to be a symmetrical dimeric derivative. The above data suggested **3** was a symmetrical dimer of **8**, connecting at C-3/C-10 between the two units (Figure 2), which was further confirmed by the HMBC correlation from H-3 to C-10. Thus, the planar structure of **3** was confirmed as shown (Figure 1), and named flavimycin C. The ^1^H and ^13^C NMR spectra (Table 1 and Table 2) of this aromatic polyketide dimer only exhibited a set of signals of aromatic polyketide monomer. There were three possible absolute configurations of two chiral carbons C-3 and C-10 in **3**. The obvious negative optical activity (${\left[\alpha \right]}_{D}^{20\;}$= −70.0) and the Cotton effect indicated that compound **3** was not a mesomer, which implied the possibility of 3*R*, 10*S* was excluded. Consequently, the absolute configurations of C-3 and C-10 were the same (3*S*, 10*S* or 3*R*, 10*R*). The absolute configuration of **3** was understood to be 3*R*, 10*R* by comparing the experimental and simulated ECD curves (Figure 3).

The known compounds: epicocconigrone A (**4**) [24]; 2-(10-formyl-11,13-dihydroxy-12-methoxy-14-methyl)-6,7-dihydroxy-5-methyl-4-benzofurancarboxaldehyde (**5**) [25]; epicoccolide B (**6**) [26]; eleganketal A (**7**) [27]; 1,3-dihydro-5-methoxy-7-methylisobenzofuran (**8**) [28]; and 2,3,4-trihydroxy-6-(hydroxymethyl)-5-methylbenzyl-alcohol (**9**) [29] were identified by comparing their NMR data with those reported in the literature.

**Scheme 1:**
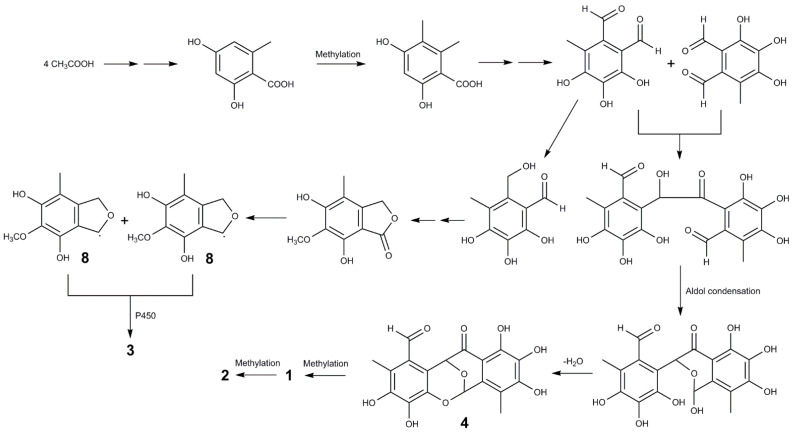
Putative biosynthetic pathways toward the formation of compounds **1**–**3**.

The new compounds **1**–**3** are all aromatic polyketide dimers, particularly compounds **1** and **2** simultaneously featuring consistent 6/6/6/6 heterotetracyclic ring cores and compounds **1**–**3** co-occurrence in the same marine-derived fungus suggest that they should originate from the same biogenetic pathway. A plausible biosynthetic pathway toward the formation of compounds **1**–**3** can be proposed by detailed analysis of their structures (Scheme 1).

### 2.2. In Vitro Evaluation of α-Glucosidase Inhibitory Activity

All compounds were tested for their *α*-glucosidase inhibitory activities using a reported method [30], with acarbose as the positive control. The results revealed that compounds **1**, **4**–**7**, and **9** showed more potent inhibitory activity (IC_50_ values ranging from 17.04 ± 0.28 to 292.47 ± 5.87 μM) than acarbose (IC_50_, 822.97 ± 7.10 μM) (Table 3). The potent *α*-glucosidase inhibitory activity of epicocconigrone A (**4**) and epicoccolide B (**6**) has been already reported [31]. It could be noted herein that the number of hydroxyl groups of polyhydroxy phenolic compounds was important for *α*-glucosidase inhibitory activity, as reflected by the low IC_50_ values of compounds **4** and **6**, while structures with fewer hydroxyl groups (compounds **1** and **5**) exhibited little activity.

## 3. Materials and Methods

### 3.1. Fungal Material and Fermentation

The fungal strain *A*. *insulicola* was isolated from deep-sea sediments, which were collected from the South China Sea at the depth of 2500 m. After grinding, the sample (1.0 g) was diluted to 10^−2^ g/mL with sterile H_2_O, 100 μL of which was spread on potato dextrose agar medium (200.0 g potato, 20.0 g glucose, and 20.0 g agar per liter of seawater) plates containing chloramphenicol as a bacterial inhibitor. It was identified by its morphological characteristics and ITS gene sequences (GenBank accessing No. ON413861), the used primers of which were ITS1 (TCCGTAGGTGAACCTGCGG) and ITS4 (TCCTCCGCTTATTGATATGC). A reference culture of *A*. *insulicola* was deposited at the Hainan Provincial Key Laboratory for Functional Components Research and Utilization of Marine Bio-resources, Haikou, China.

### 3.2. Culture Conditions

The fungal strain *A*. *insulicola* was cultured in potato dextrose broth medium (consisting of 200.0 g/L potato, 20.0 g/L glucose, and 1000.0 mL deionized water), and incubated on a rotary shaker (150 rpm) for 72 h at 28 °C. Thereafter, 3 mL of seed broth was transferred to fifty 1000 mL Erlenmeyer flasks containing solid rice medium (each flask contained 80 g rice and 120 mL seawater), used for fermentation. The flasks were incubated under static conditions at room temperature for 30 days.

### 3.3. General Experimental Procedures

Optical rotation was measured using a Modular Circular Polarimeter 5100 polarimeter (Anton Paar, Austria). The NMR spectra were measured on Bruker Avance 500 NMR spectrometer (Bruker, Bremen, Germany) and Bruker DRX-600 spectrometer (Bruker Biospin AG, Fällanden, Germany) using TMS as an internal standard. HRESIMS were determined with an API QSTAR Pulsar mass spectrometer (Bruker, Bremen, Germany). ECD and UV spectra were recorded on a MOS-500 spectrometer (Biologic, France). IR data were measured on a Nicolet 380 infrared spectrometer (Thermo Electron Corporation, Madison, WI, USA). Analytic HPLC was performed with an Agilent Technologies 1260 Infinity II equipped with an Agilent DAD G1315D detector (Agilent, Palo Alto, CA, USA), the separation columns were (COSMOSIL-packed C_18_, 5 mm, 4.6 mm × 250 mm). Semi-preparative HPLC was performed on reversed-phased columns (COSMOSIL-packed C_18_, 5 mm, 10 mm × 250 mm). Silica gel (60–80, 200–300 and 300–400 mesh, Qingdao Marine Chemical Co. Ltd., Qingdao, China) and Sephadex LH-20 (Merck, Germany) were used for column chromatography. TLC was conducted on precoated silica gel GF254 plates (Qingdao Marine Chemical Co. Ltd., Qingdao, China), and spots were detected by spraying with 10% H_2_SO_4_ in EtOH followed by heating.

### 3.4. Extraction and Isolation

After the fermentation of the strain, the cultures were extracted with EtOAc, then filtered with filter paper. After repeating the procedure three times, the EtOAc extract was evaporated under a reduced pressure to obtain a crude extract (124.0 g). The crude extract was dispersed in water and extracted with petroleum ether, ethyl acetate and *n*-butanol three times, respectively. After vacuum concentration, the petroleum ether extract (11.3 g), ethyl acetate extract (34.0 g) and n-butanol extract (20.0 g) were obtained, respectively. Then, the EtOAc extract (34.0 g) was subjected to silica gel vacuum liquid chromatography using step gradient elution with CHCl_3_/MeOH (1:0, 200:1, 150:1, 100:1, 80:1, 50:1, 20:1, 10:1, 0:1, *v*/*v*) to obtain 13 fractions (Fr.1–Fr.13). Fr.4 (425.0 mg) was applied to Sephadex LH-20 gel chromatography eluted with CHCl_3_/MeOH (1:1, *v*/*v*) to give six subfractions (Fr.4.1–Fr.4.6). Fr.4.3 (150.5 mg) was subjected to silica gel column chromatography (petroleum ether/EtOAc, 10:1, *v*/*v*) to afford nine subfractions (Fr.4.3.1–Fr.4.3.9). Fr.4.3.9 (40.5 mg) was separated by semi-preparative HPLC, eluting with 45% MeOH/H_2_O to yield compound **2** (*t*_R_ 11.5 min, 4.5 mg), and Fr.4.3.7 (29.8 mg) was separated by semi-preparative HPLC, eluting with 35% MeOH/H_2_O to give compound **8** (*t*_R_ 15.3 min, 4.2 mg). Fr.6 (1.15 g) was applied to ODS chromatography eluting with MeOH/H_2_O (10%–100%) to give thirteen subfractions (Fr.6.1–Fr.6.13). Fr.6.11 (91.5 mg) was subjected to Sephadex LH-20 (eluted with 100% MeOH) and then purified by semi-preparative HPLC (eluted with 48% MeOH/H_2_O) to give compound **1** (*t*_R_ 21.9 min, 11.1 mg). Fr.6.12 (58.9 mg) was subjected to Sephadex LH-20 (eluted with 100% MeOH) and then purified by semi-preparative HPLC (eluted with 65% MeOH/H_2_O) to give compound **5** (*t*_R_ 10.0 min, 4.2 mg). Fr.6.6 (72.3 mg) was purified on silica gel (petroleum ether/EtOAc, 3:2, *v*/*v*) to yield compound **3** (7.5 mg). Fr.9 (10.0 g) was subjected to Sephadex LH-20 gel chromatography eluted with MeOH to give ten subfractions (Fr.9.1–Fr.9.10). Fr.9.7 (2.1 g) was subjected to silica gel column chromatography (CH_2_Cl_2_/MeOH, 100:1, *v*/*v*), and subsequently purified by semi-preparative HPLC, eluting with 50 % MeOH/H_2_O to yield compounds **4** (*t*_R_ 12.0 min, 5.1 mg) and **6** (*t*_R_ 18.5 min, 2.7 mg). Fr.9.6 (1.67 g) was separated by Sephadex LH-20 column chromatography eluted with MeOH and then purified by silica gel column chromatography eluting with petroleum ether/EtOAc (3:1; *v*/*v*) to obtain compound **9** (5.1 mg). Fr.9.8 (0.8 g) was subjected to silica gel column chromatography (CH_2_Cl_2_/MeOH, 35:1, *v*/*v*), and subsequently purified by semi-preparative HPLC, eluting with 55 % MeOH/H_2_O to yield compound **7** (*t*_R_ 6.8 min, 8.1 mg).

Epicocconigrone C (**1**): Yellow film. ${\left[\alpha \right]}_{D}^{20}\;$= +98.0 (*c* 0.10, MeOH); UV (MeOH) *λ*_max_ (log*ε*): 237 (4.31) nm; 261 (3.91) nm; 309 (4.27) nm; 359 (4.06) nm; IR (KBr) *v*_max_ (cm^−1^): 3413, 1669, 1466, 1395, 1355, 1296, 1117. ^1^H and ^13^C NMR data see Table 1 and Table 2; HRESIMS [M + Na]^+^ *m/z* 411.0698 (calcd. for C_19_H_16_O_9_Na, 411.0687).

Epicocconigrone D (**2**): Yellow film. ${\left[\alpha \right]}_{D}^{20\;}$= +57.0 (*c* 0.10, MeOH); UV (MeOH) *λ*_max_ (log*ε*): 234 (4.26) nm; 260 (4.04) nm; 309 (4.21) nm; 365 (4.19) nm; IR (KBr) *v*_max_ (cm^−1^): 3446, 2931, 1626, 1469, 1359, 1226, 1154, 1115. ^1^H and ^13^C NMR data see Table 1 and Table 2; HRESIMS [M + Na]^+^ *m/z* 427.1004 (calcd. for C_20_H_20_O_9_Na, 427.1000).

Flavimycin C (**3**): White film. ${\left[\alpha \right]}_{D}^{20\;}$= −70.0 (*c* 0.10, MeOH); UV (MeOH) *λ*_max_ (log*ε*): 232 (4.15) nm; 284 (3.56) nm; IR (KBr) *v*_max_ (cm^−1^): 3449, 2928, 1606, 1478, 1376, 1264, 1110, 1027. ^1^H and ^13^C NMR data see Table 1 and Table 2; HRESIMS [M + H]^+^ *m/z* 391.1385 (calcd. for C_20_H_23_O_8_, 391.1387).

### 3.5. ECD Calculation

The conformers of compounds were generated using the Confab [32] program ebbed in the Openbabel 3.1.1 software, and further optimized with xtb at GFN2 level [33]. The conformers with population over 1% were subjected to geometry optimization using the Gaussian 16 package [34] at B3LYP/6-31G(d) level and proceeded to calculation of excitation energies, oscillator strength, and rotatory strength at B3LYP/TZVP level in the polarizable continuum model (PCM, methanol). The ECD spectra were Boltzmann-weighted and generated using SpecDis 1.71 software [35].

### 3.6. α-Glucosidase Inhibitory Activity

All the assays were carried out under 0.1 M sodium phosphate buffer (PH = 6.8). The samples were dissolved with DMSO and diluted into a series of gradient concentrations (final concentrations of 6.25, 12.5, 25, 50, 100, 200, 400, and 800 μM). The 10 μL sample was mixed with 100 μL α-glucosidase solution (0.2 U/mL, Sigma) and shaken well, then added to a 96-well plate and placed at 37 °C for 15 min. Subsequently, 40 μL of 2.5 mM 4-nitrophenyl-α-D-glucopyranoside was added and further incubated at 37 °C for 15 min. Finally, the OD value of each well was detected at 405 nm wavelength of microplate reader. Acarbose was used as a positive control. The control was prepared by adding DMSO instead of the sample in the same way as the test. The blank was prepared by adding sodium phosphate buffer instead of 4-nitrophenyl-α-D-glucopyranoside using the same method. The percentage inhibition was calculated using the following equation:


% inhibition= [(OD_control_ − OD_sample_)/(OD_control_ − OD_blank_)] × 100

## 4. Conclusions

In summary, two new tetracyclic cores of integrastatins, named epicocconigrones C–D (**1**–**2**), one new dimeric isobenzofuran, named flavimycin C (**3**), and six known compounds (**4**–**9**) were isolated from fermentation cultures of the deep-sea sediment-derived fungus *A*. *insulicola*. The biological evaluation revealed compounds **1**, **4**–**7**, **9** exhibited significant α-glucosidase inhibitory with IC_50_ values ranging from 17.04 ± 0.28 to 292.47 ± 5.87 μM, among which compound **6** was the most potent α-glucosidase inhibitor, with an IC_50_ value 48-fold stronger than positive control acarbose. Comparing the structure of compounds **1**, **4**, **5** and **6** revealed the α-glucosidase inhibitory activity was greatly enhanced after the hydroxyl group replaced the methoxy group, which further confirmed that polyhydroxy phenolic compounds were efficient α-glucosidase inhibitors, and provided a reference value for the synthesis of novel α-glucosidase inhibitors. In conclusion, the study has enriched the structural diversity of phenolic compounds and provided a promising lead toward the development of novel α-glucosidase inhibitors.

## Figures and Tables

**Table 1:** ^1^H NMR data of epicocconigrones C–D (**1**–**2**) and flavimycin C (**3**) (*δ* in ppm, *J* in Hz) in DMSO-*d*_6_.

Position	1 *^a^*	2 *^a^*	3 *^b^*
1			4.54 (d, 15.0)
			4.65 (d, 15.0)
2	6.83, s	6.76, s	
3			4.30, s
10	6.38, s	5.65, s	4.30, s
12			4.54 (d, 15.0)
			4.65 (d, 15.0)
17	2.31, s	2.09, s	
18	10.34, s	4.81 (d, 12.1)	
		4.32 (d, 12.1)	
19	2.26, s	2.18, s	1.88, s
20			1.88, s
6-OCH_3_	3.70, s	3.57, s	3.64, s
14-OCH_3_			3.64, s
15-OCH_3_		3.69, s	
5-OH			8.55, s
7-OH	11.33, s	11.46, s	8.68, s
14-OH		8.92, s	
15-OH			8.68, s
17-OH			8.55, s

*^a^* Recorded at 500 MHz; *^b^* Recorded at 600 MHz.

**Table 2:** ^13^C NMR (125 MHz) data of epicocconigrones C-D (**1**–**2**) and flavimycin C (**3**) in DMSO-*d*_6_.

Position	1	2	3
1			65.8, CH_2_
2	89.8, CH	89.9, CH	
3	130.8, C	131.0, C	66.1, CH
4	115.8, C	115.8, C	112.2, C
5	156.9, C	158.9, C	147.0, C
6	134.7, C	134.6, C	134.3, C
7	153.6, C	153.6, C	147.8, C
8	104.5, C	103.4, C	109.7, C
9	196.9, C	196.8, C	129.7, C
10	68.6, CH	70.3, CH	66.1, CH
11	112.6, C	108.3, C	
12	121.7, C	132.1, C	65.8, CH_2_
13	121.9, C	118.1, C	129.7, C
14	144.3, C	148.5, C	109.7, C
15	138.4, C	134.6, C	147.8, C
16	135.8, C	140.3, C	134.3, C
17	11.8, CH_3_	11.0, CH_3_	147.0, C
18	191.2, CH	55.8, CH_2_	112.2, C
19	10.2, CH_3_	10.3, CH_3_	9.5, CH_3_
20			9.5, CH_3_
6-OCH_3_	60.2, CH_3_	60.3, CH_3_	60.2, CH_3_
14-OCH_3_			60.2, CH_3_
15-OCH_3_		60.0, CH_3_	

**Table 3:** *α*-Glucosidase inhibitory activities of compounds **1**–**9**.

Compounds	IC_50_ ± SD (μM) ^a^
**1**	292.47 ± 5.87
**2**	–
**3**	–
**4**	25.69 ± 0.30
**5**	40.07 ± 4.64
**6**	17.04 ± 0.28
**7**	49.53 ± 2.45
**8**	–
**9**	130.63 ± 2.87
Acarbose ^b^	822.97 ± 7.10

^a^ Values represent means ± SD based on three parallel experiments. ^b^ Positive control.–no activity at a concentration of 200 μM.

## Data Availability

The authors declare that all relevant data supporting the results of this study are available within the article and its Supplementary Materials file, or from the corresponding authors upon request.

## Supplementary Materials

The following supporting information can be downloaded at: https://www.mdpi.com/article/10.3390/md21030157/s1, Figures S1–S30: 1D, 2D NMR, MS, UV, and IR spectra of compounds **1**–**3**.
