# Supplementary material for: Potential α-Glucosidase Inhibitors from the Deep-Sea Sediment-Derived Fungus Aspergillus insulicola
Source: Mar Drugs. 2023 Feb 26;21(3):157. doi: 10.3390/md21030157 (PMC10056930; doi:10.3390/md21030157)

Supporting Information

Potential α-Glucosidase Inhibitors from the Deep-Sea Sediment-Derived Fungus *Aspergillus insulicola*

Weibo Zhao [1,2,†], Yanbo Zeng [2,3,†], Wenjun Chang [2,3], Huiqin Chen [2], Hao Wang [2], Haofu Dai [2], and Fang Lv [1,*]

[1] Beijing Key Laboratory for Separation and Analysis in Biomedicine and Pharmaceuticals, School of Life Science, Beijing Institute of Technology, Beijing, 100081, China

[2] Hainan Provincial Key Laboratory for Functional Components Research and Utilization of Marine Bio-resources , Institute of Tropical Bioscience and Biotechnology, Chinese Academy of Tropical Agricultural Sciences & Key Laboratory for Biology and Genetic Resources of Tropical Crops of Hainan Province, Hainan Institute for Tropical Agricultural Resources, Haikou 571101, China

[3] Zhanjiang Experimental Station of Chinese Academy of Tropical Agricultural Sciences, Zhanjiang, 524013, China

***** Correspondence: lvfangbeijing@bit.edu.cn (F.L.)

† These authors contributed equally to this work.

Figure S1. ^{1}H NMR (500 MHz, DMSO-d_6) spectrum of compound **1**

Figure S2. ^{13}C NMR (125 MHz, DMSO-d_6) spectrum of compound **1**

Figure S3. The DEPT spectrum of compound **1** in DMSO-d_6

Figure S4. The HSQC spectrum of compound **1** in DMSO-d_6

Figure S5. The HMBC spectrum of compound **1** in DMSO-d_6

Figure S6. The COSY spectrum of compound **1** in DMSO-d_6

Figure S7. The NOESY spectrum of compound **1** in DMSO-d_6

Figure S8. The HRESIMS spectrum of compound **1**

Figure S9. The UV spectrum of compound **1**

Figure S10. The IR spectrum of compound **1**

Figure S11. ^{1}H NMR (500 MHz, DMSO-d_6) spectrum of compound **2**

Figure S12. ^{13}C NMR (125 MHz, DMSO-d_6) spectrum of compound **2**

Figure S13. The DEPT spectrum of compound **2** in DMSO-d_6

Figure S14. The HSQC spectrum of compound **2** in DMSO-d_6

Figure S15. The HMBC spectrum of compound **2** in DMSO-d_6

Figure S16. The COSY spectrum of compound **2** in DMSO-d_6

Figure S17. The ROESY spectrum of compound **2** in DMSO-d_6

Figure S18. The HRESIMS spectrum of compound **2**

Figure S19. The UV spectrum of compound **2**

Figure S20. The IR spectrum of compound **2**

Figure S21. ^{1}H NMR (600 MHz, DMSO-d_6) spectrum of compound **3**

Figure S22. ^{13}C NMR (150 MHz, DMSO-d_6) spectrum of compound **3**

Figure S23. The DEPT spectrum of compound **3** in DMSO-d_6

Figure S24. The HSQC spectrum of compound **3** in DMSO-d_6

Figure S25. The HMBC spectrum of compound **3** in DMSO-d_6

Figure S26. The COSY spectrum of compound **3** in DMSO-d_6

Figure S27. The ROESY spectrum of compound **3** in DMSO-d_6

Figure S28. The HRESIMS spectrum of compound **3**

Figure S29. The UV spectrum of compound **3**

Figure S30. The IR spectrum of compound **3**

Figure S1. ^{1}H NMR (500 MHz, DMSO-d_6) spectrum of compound **1**

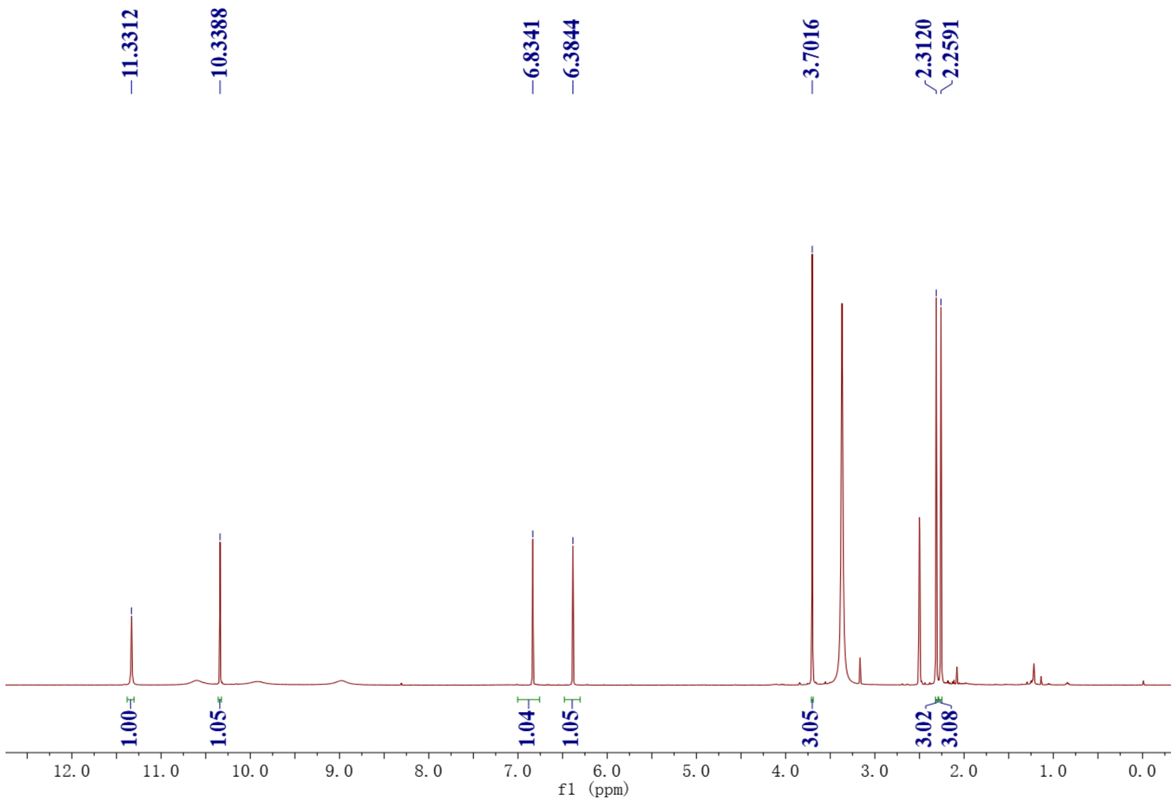

Figure S2. ^{13}C NMR (125 MHz, DMSO-d_6) spectrum of compound **1**

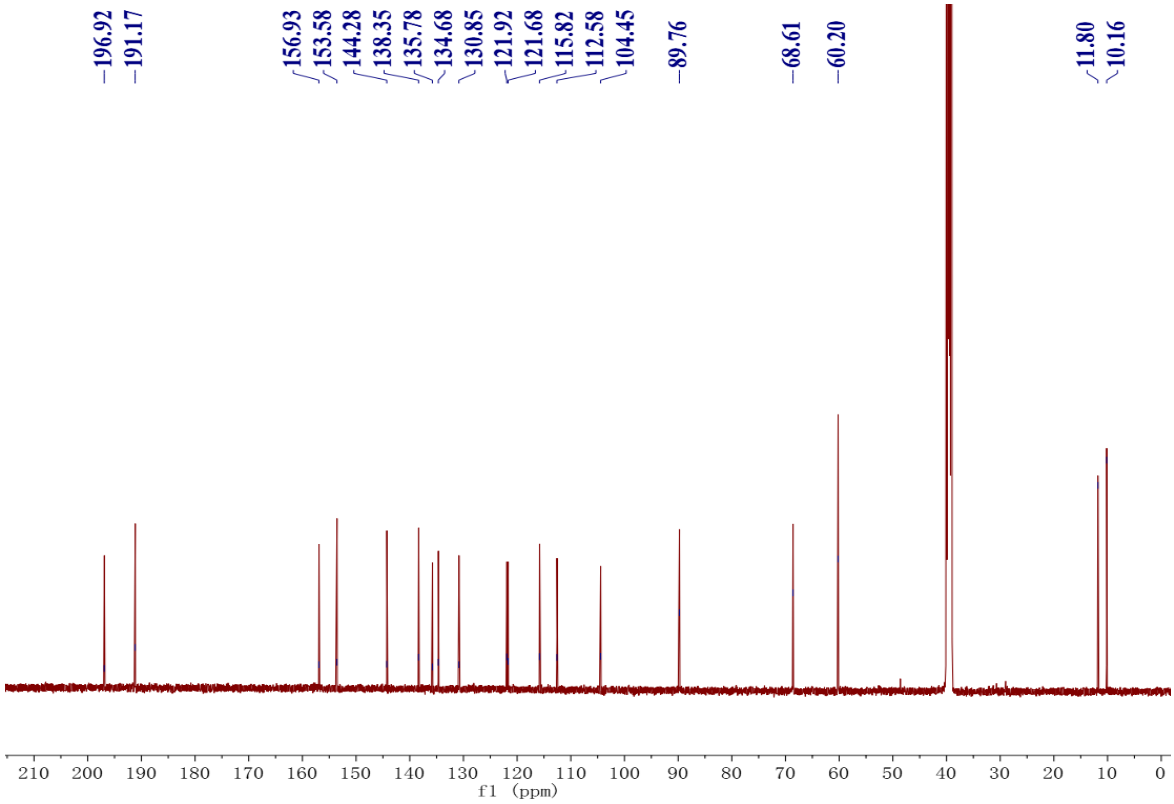

Figure S3. The DEPT spectrum of compound **1** in DMSO-d_6

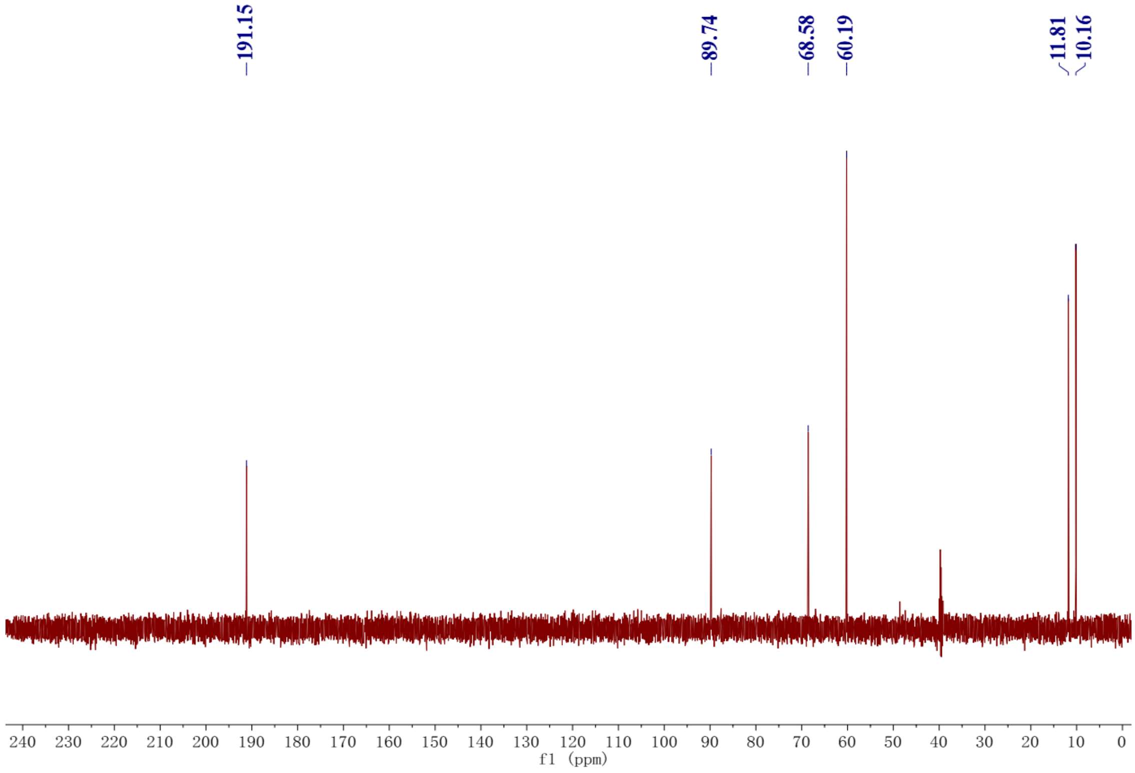

Figure S4. The HSQC spectrum of compound **1** in DMSO-d_6

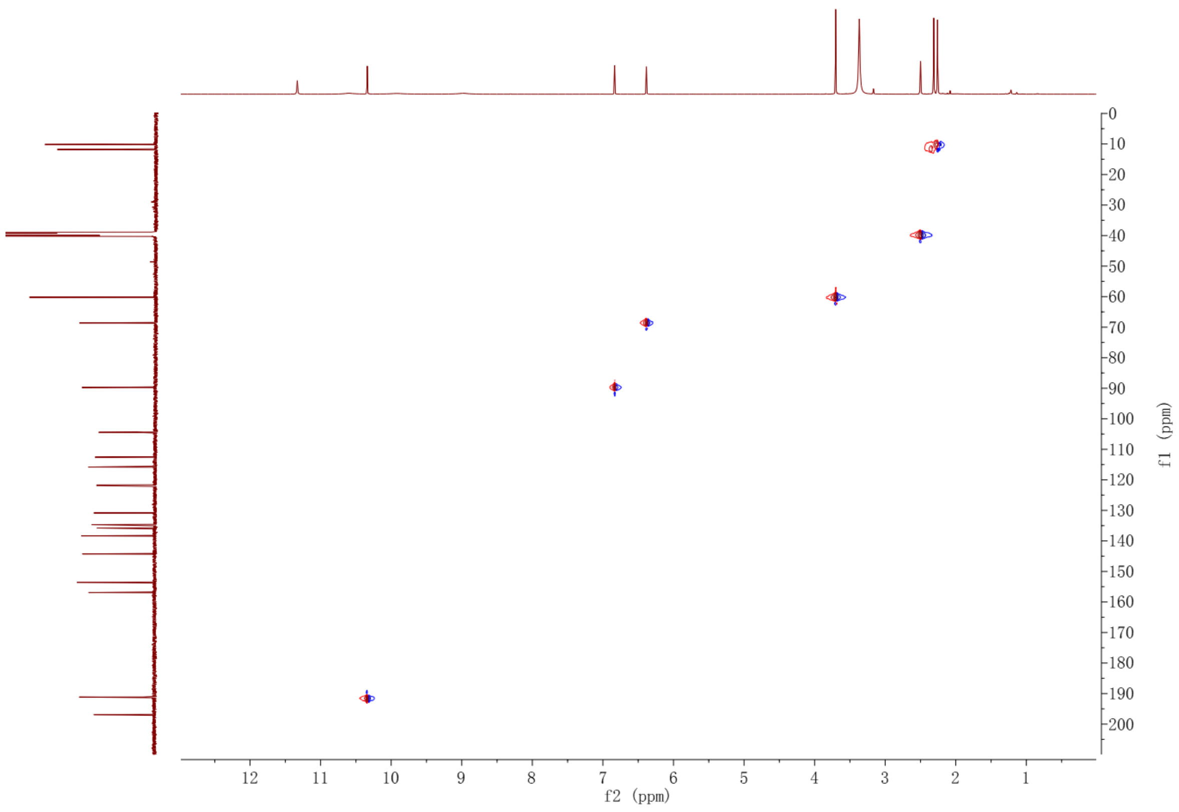

Figure S5. The HMBC spectrum of compound **1** in DMSO-d_6

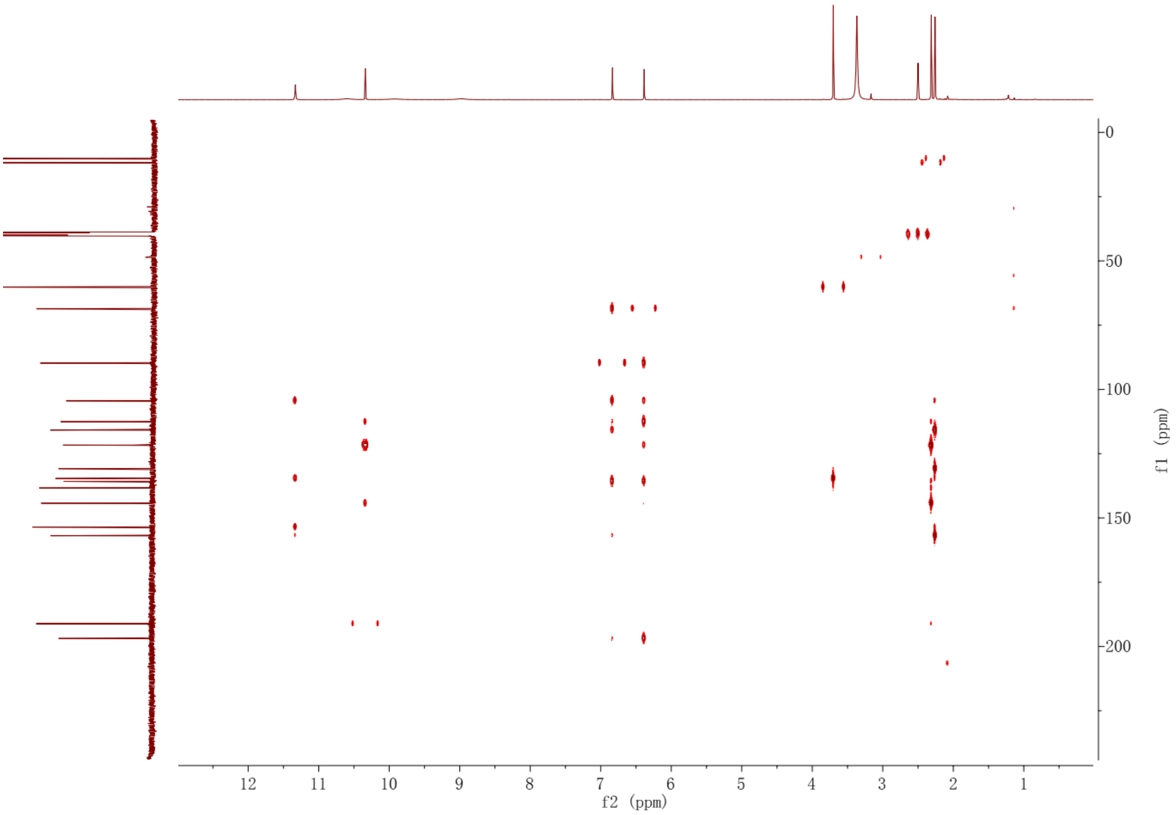

Figure S6. The COSY spectrum of compound **1** in DMSO-d_6

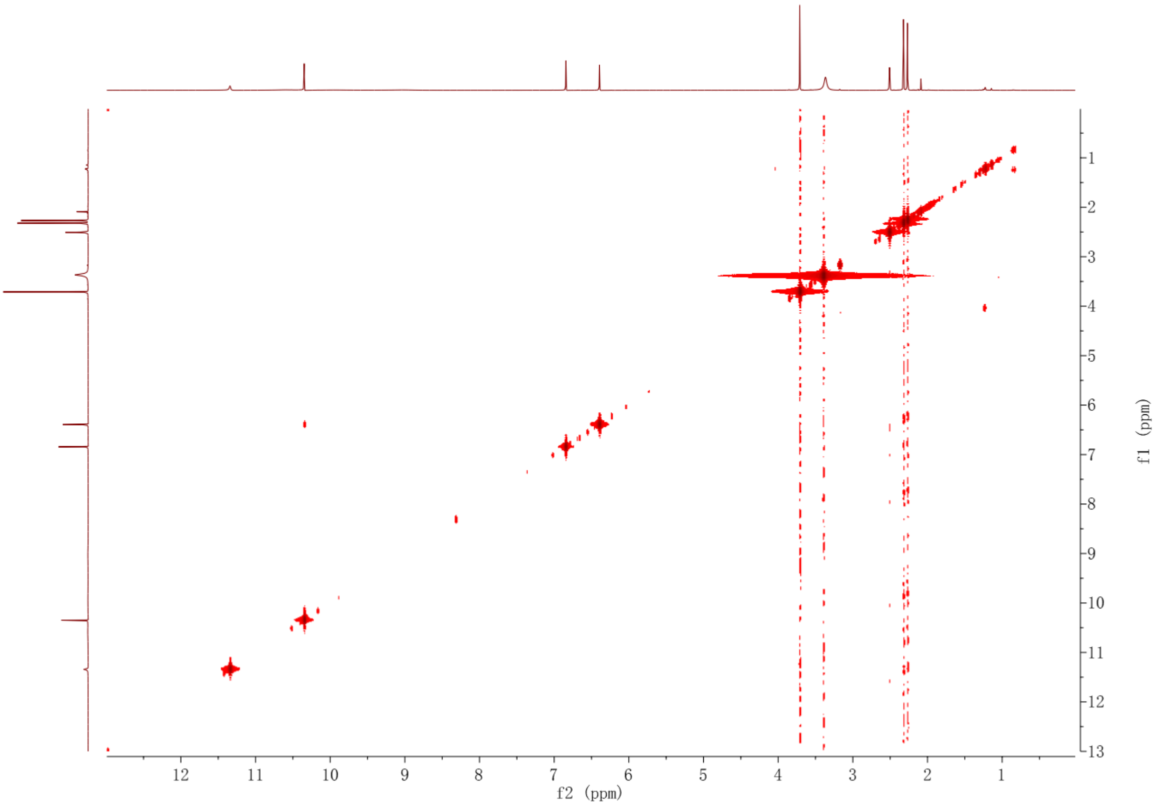

Figure S7. The NOESY spectrum of compound **1** in DMSO-d_6

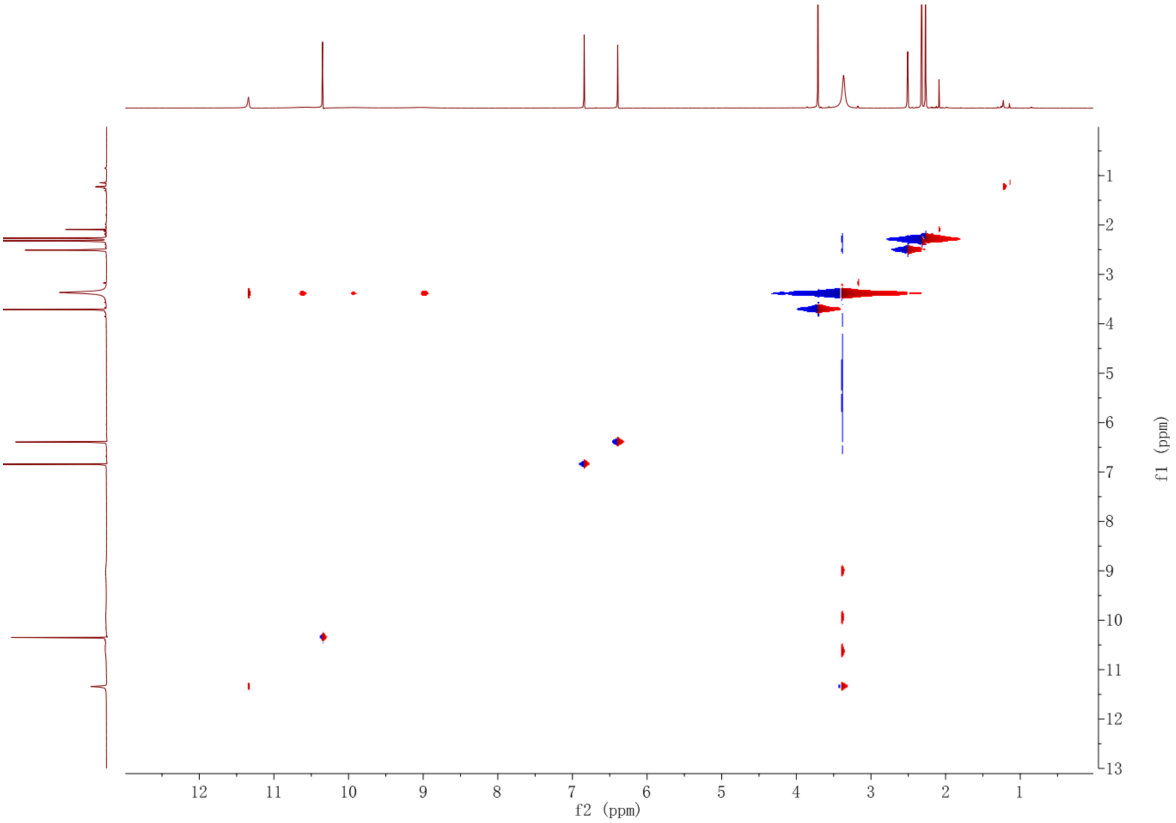

Figure S8. The HRESIMS spectrum of compound **1**

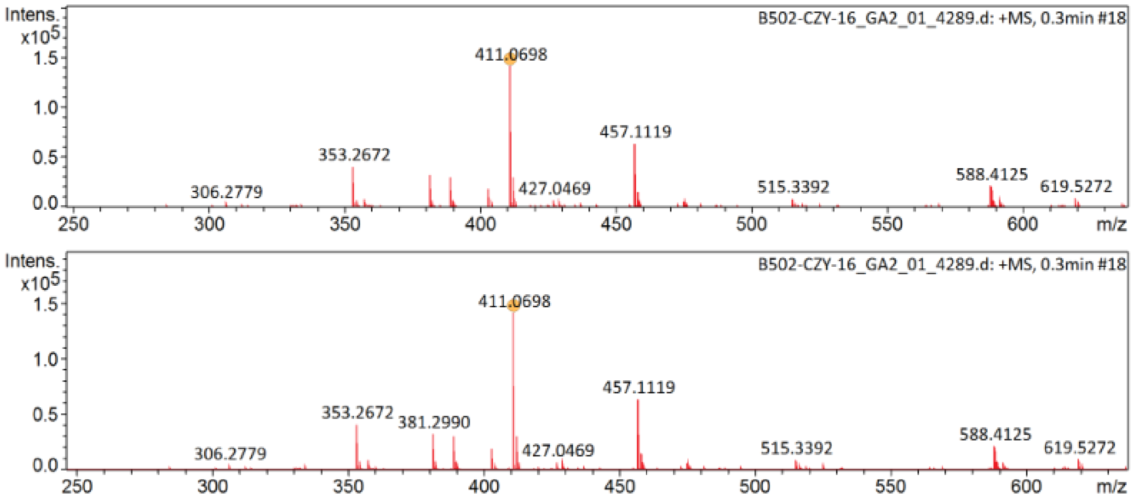

Meas. m/z	#	Ion Formula	m/z	err [ppm]	mSigma	# mSigma	Score	rdb	e⁻ Conf	N-Rule	Adduct
411.0698	1	C19H16NaO9	411.0687	-2.7	4.0	1	100.00	12.0	even	ok	M+Na

Figure S9. The UV spectrum of compound **1**

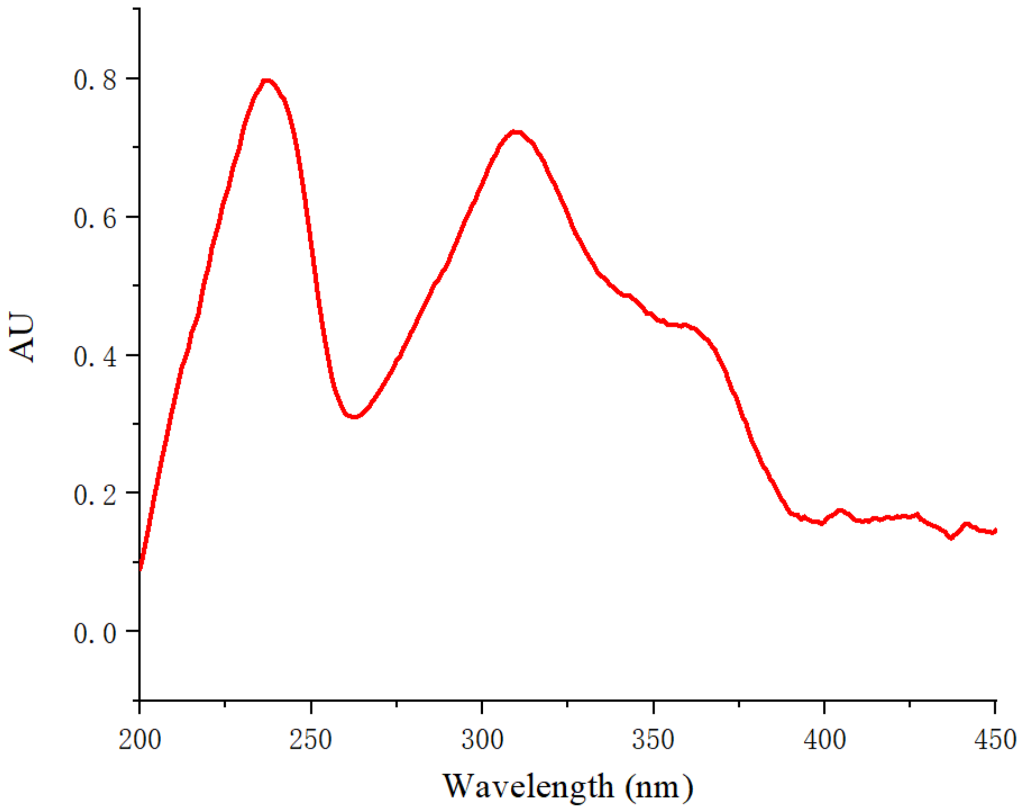

Figure S10. The IR spectrum of compound **1**

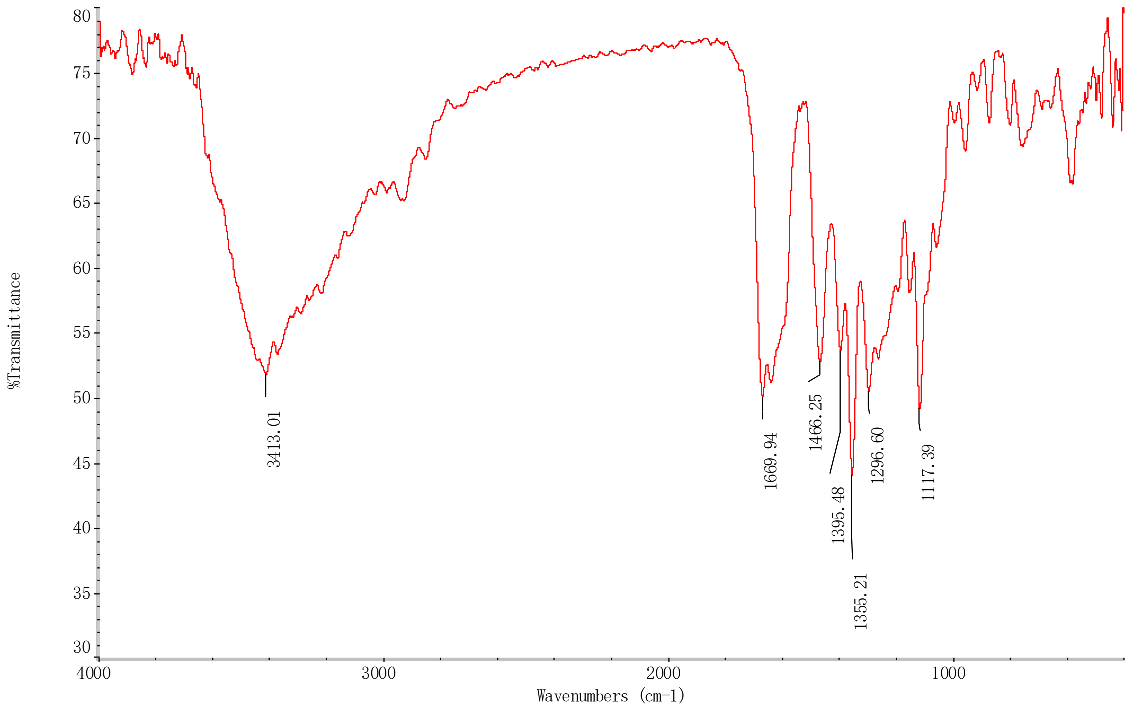

Figure S11. ^{1}H NMR (500 MHz, DMSO-d_6) spectrum of compound **2**

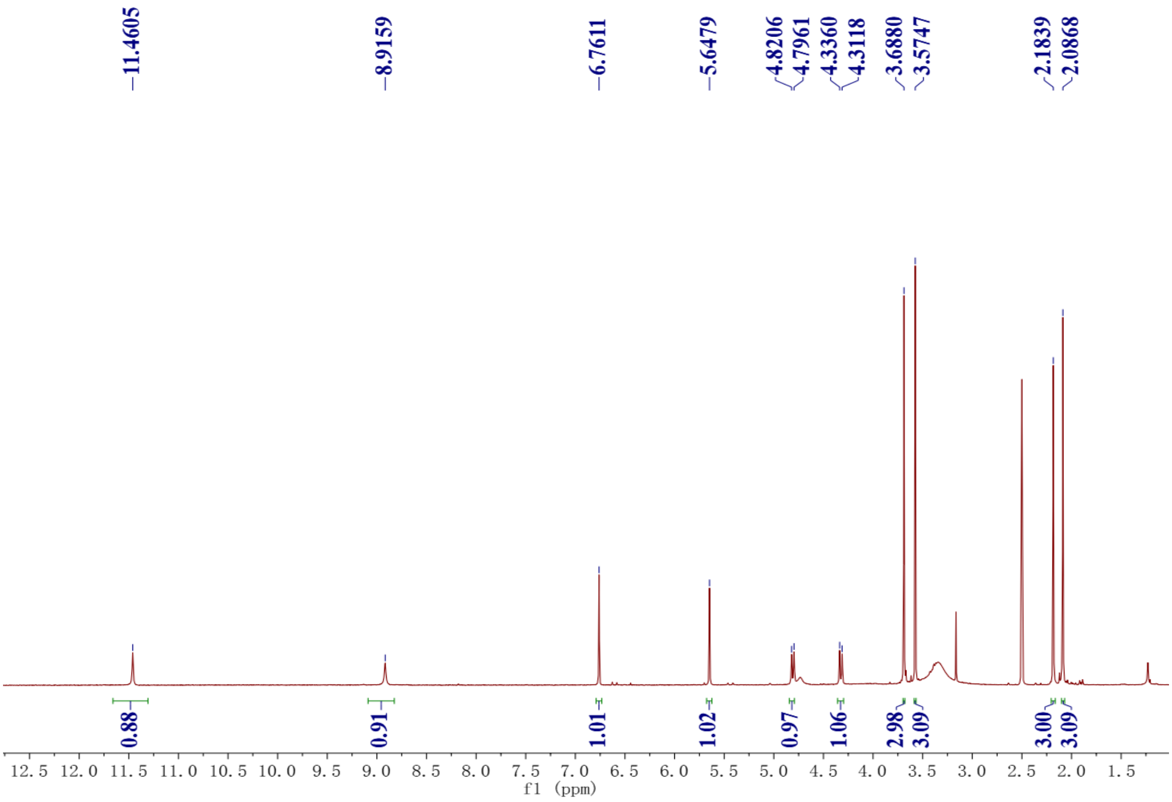

Figure S12. ^{13}C NMR (125 MHz, DMSO-d_6) spectrum of compound **2**

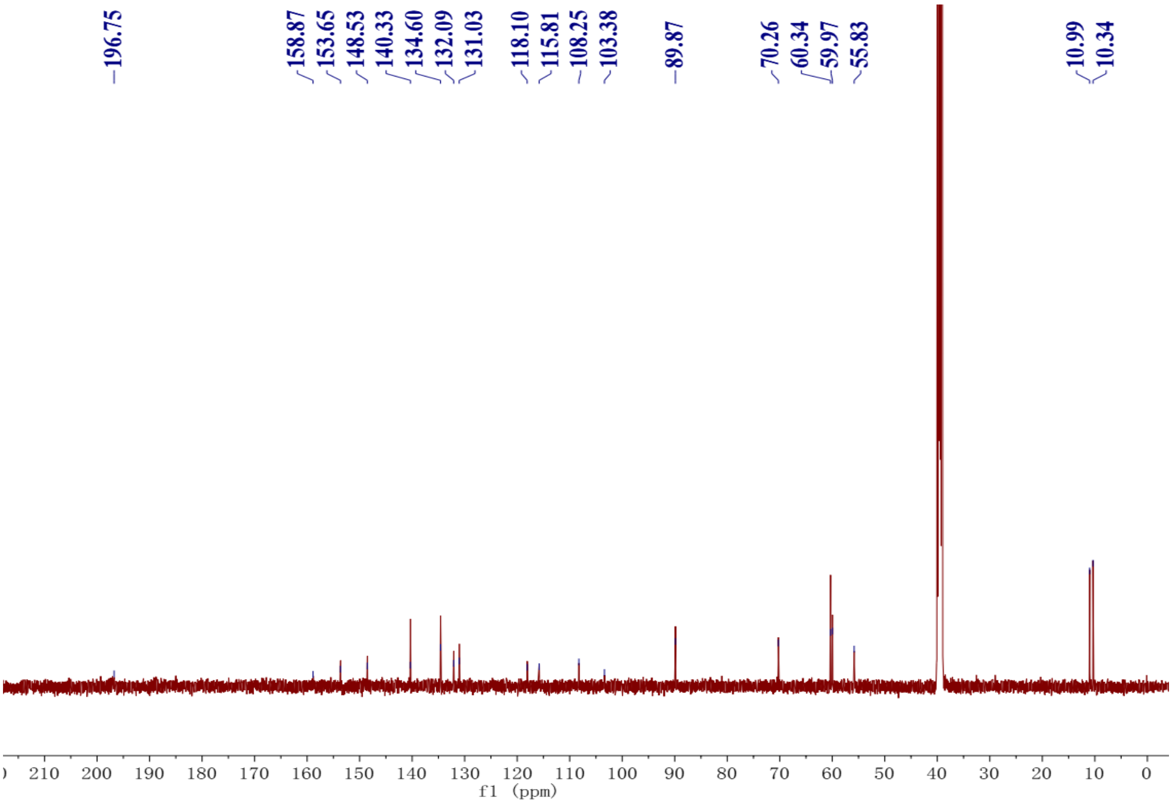

Figure S13. The DEPT spectrum of compound **2** in DMSO-d_6

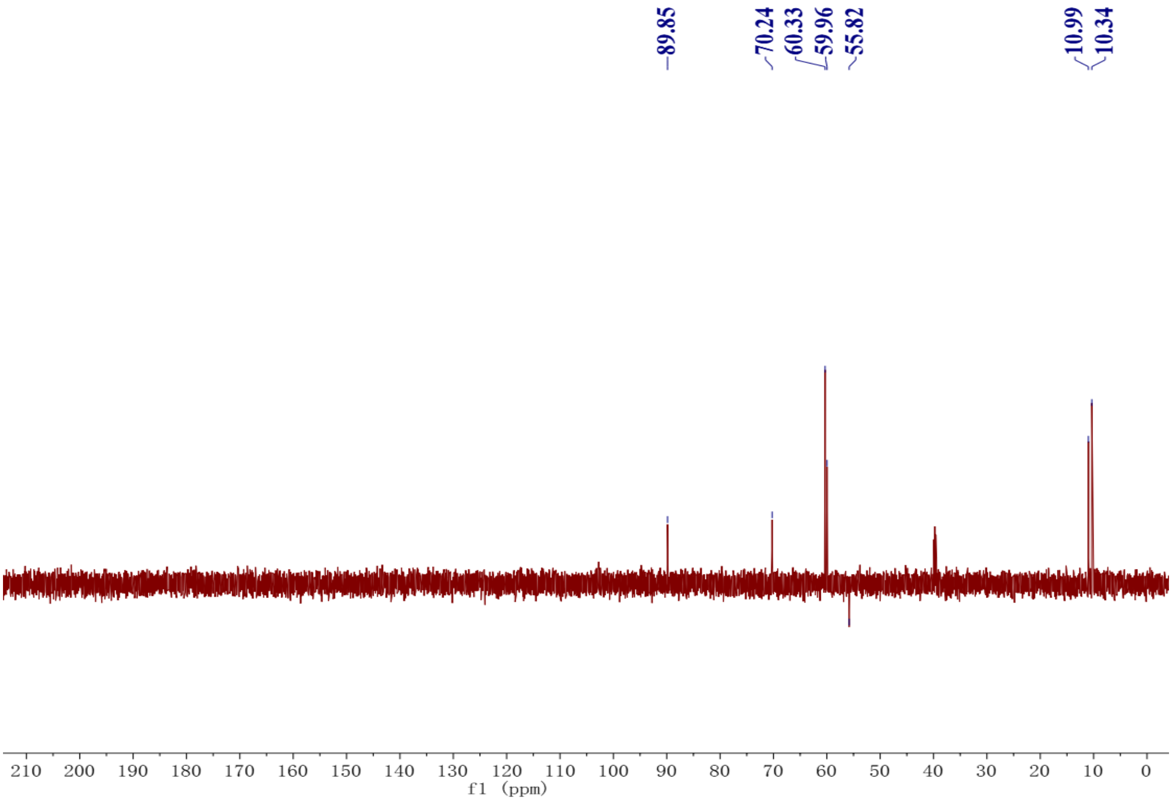

Figure S14. The HSQC spectrum of compound **2** in DMSO-d_6

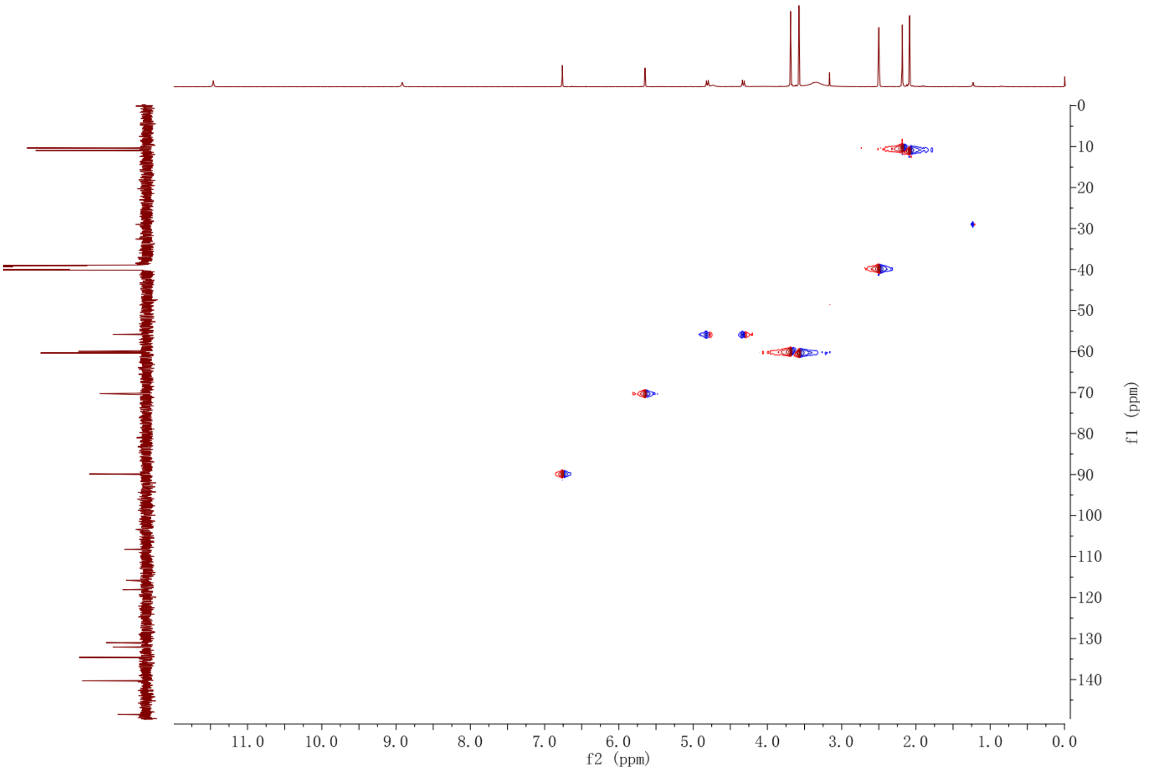

Figure S15. The HMBC spectrum of compound **2** in DMSO-d_6

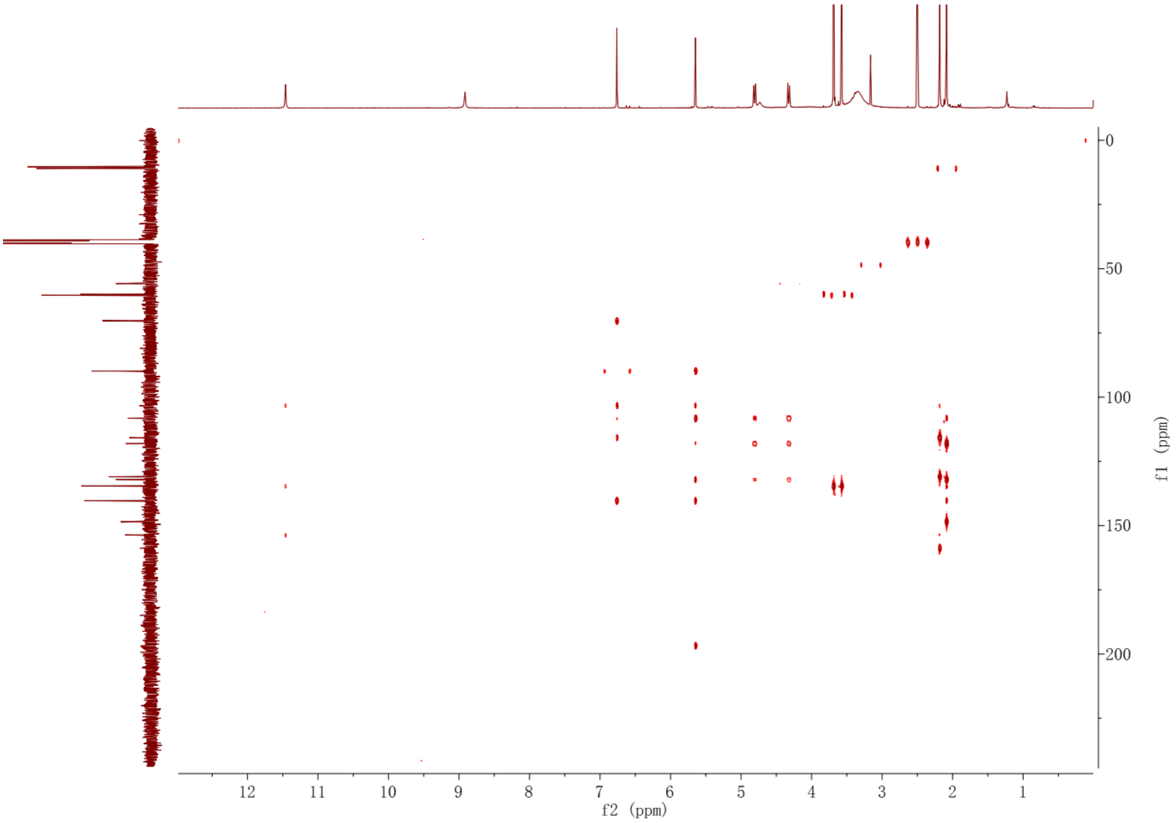

Figure S16. The COSY spectrum of compound **2** in DMSO-d_6

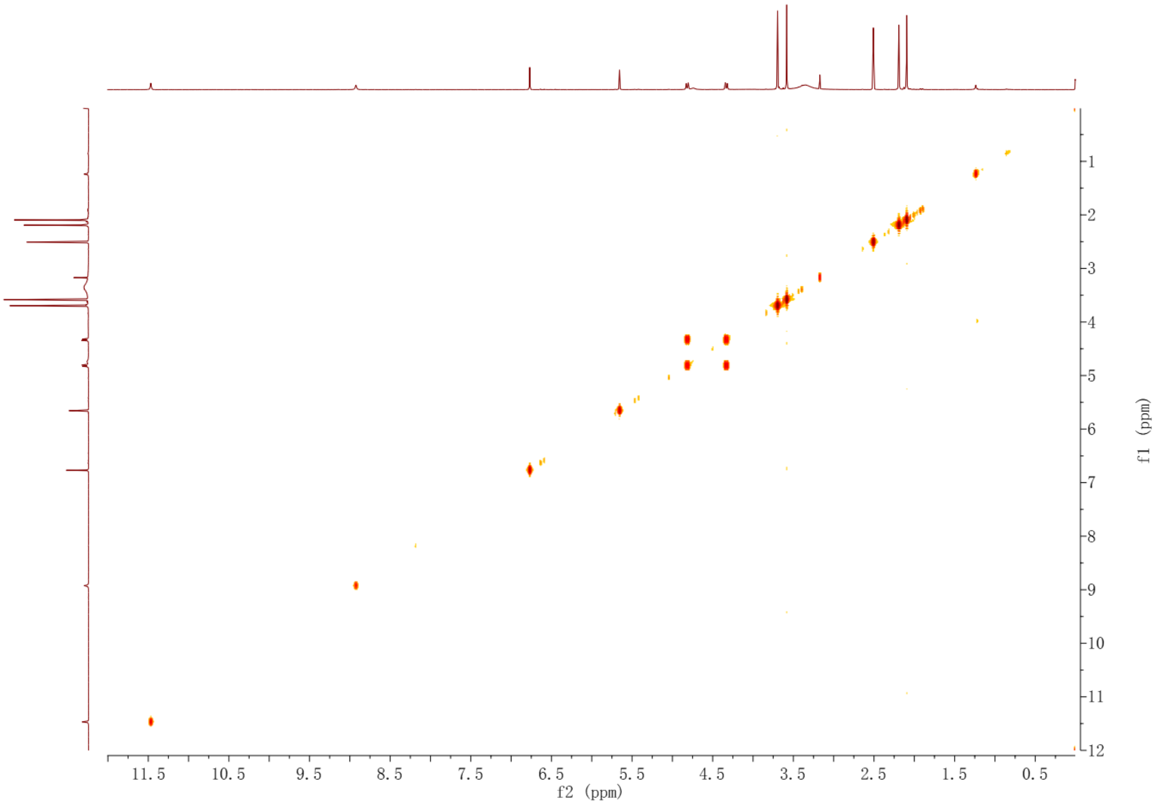

Figure S17. The ROESY spectrum of compound **2** in DMSO-d_6

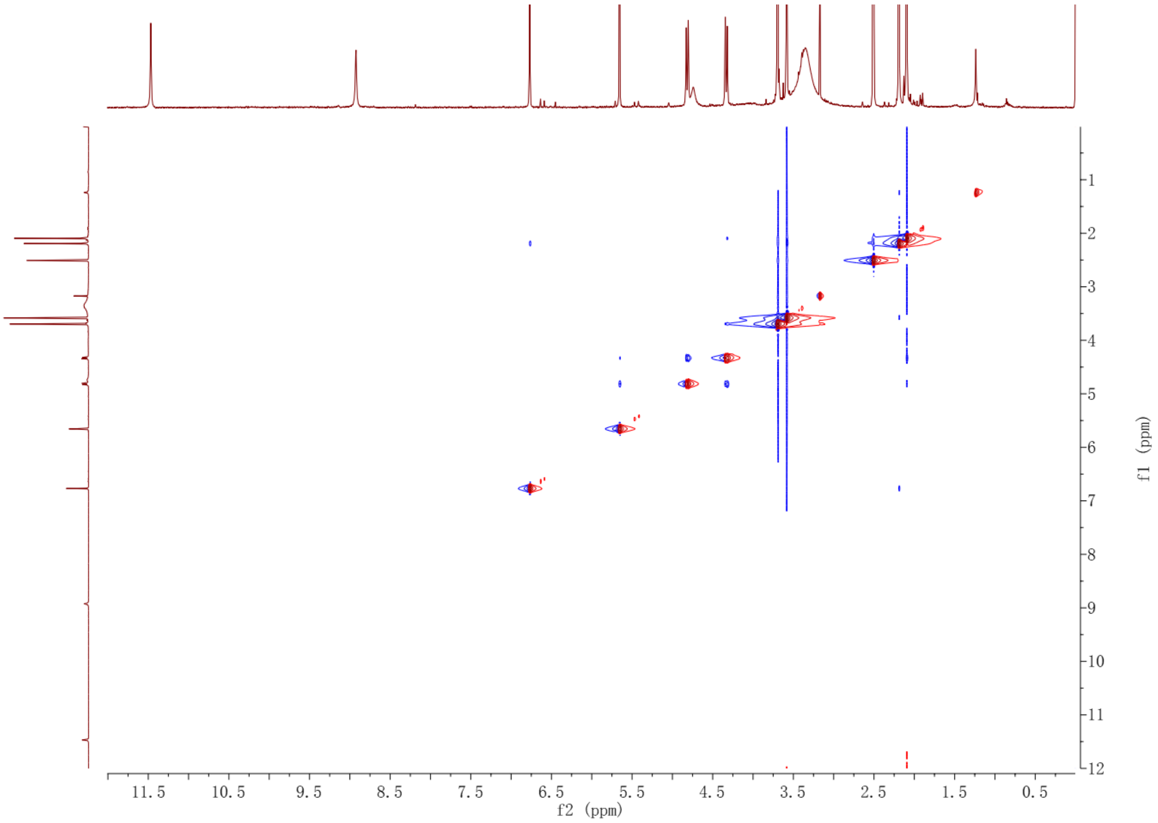

Figure S18. The HRESIMS spectrum of compound **2**

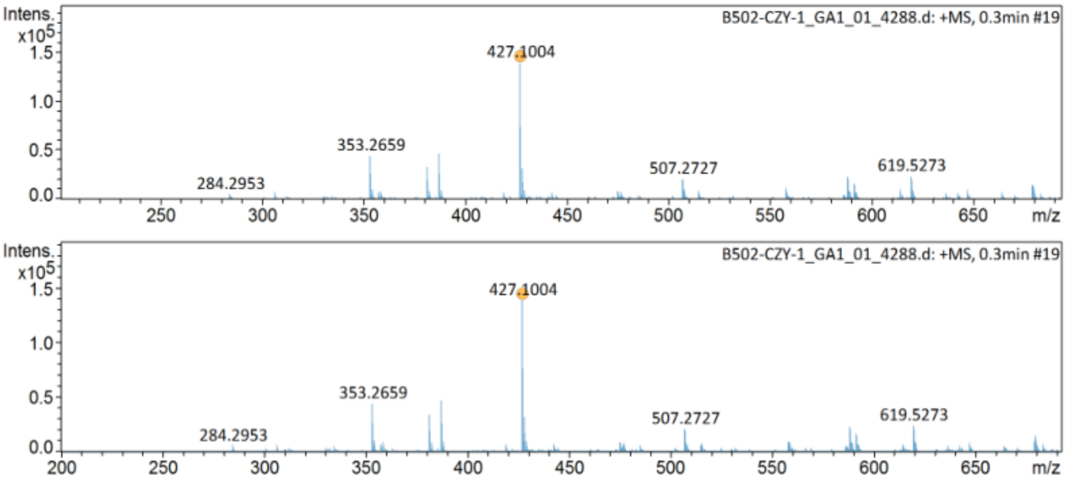

Meas. m/z	#	Ion Formula	m/z	err [ppm]	mSigma	# mSigma	Score	rdb	e⁻ Conf	N-Rule	Adduct
427.1004	1	C20H20NaO9	427.1000	-1.1	24.4	1	100.00	11.0	even	ok	M+Na

Figure S19. The UV spectrum of compound **2**

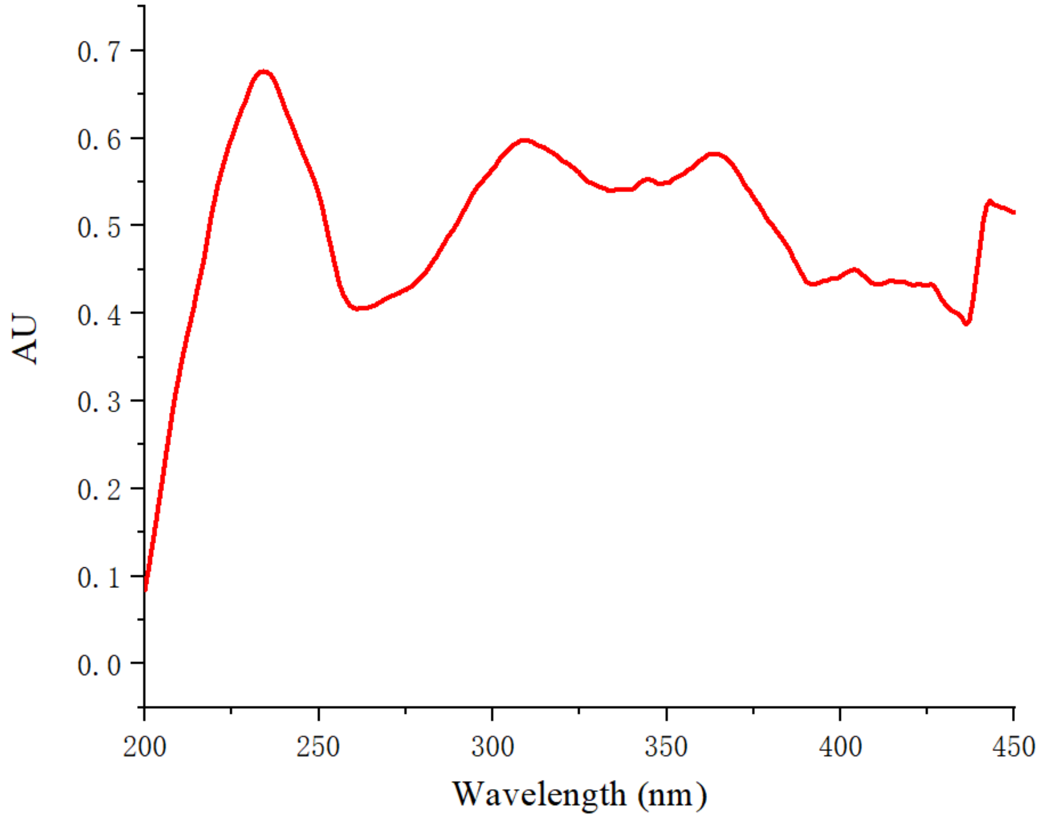

Figure S20. The IR spectrum of compound **2**

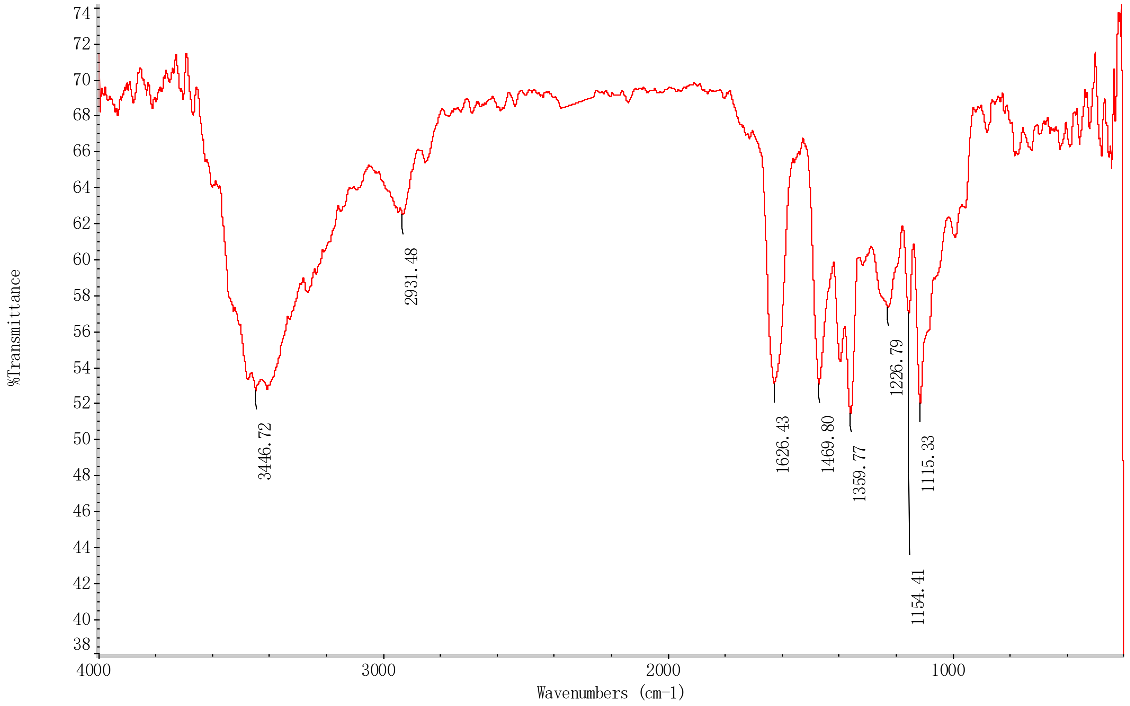

Figure S21. ^{1}H NMR (600 MHz, DMSO-d_6) spectrum of compound **3**

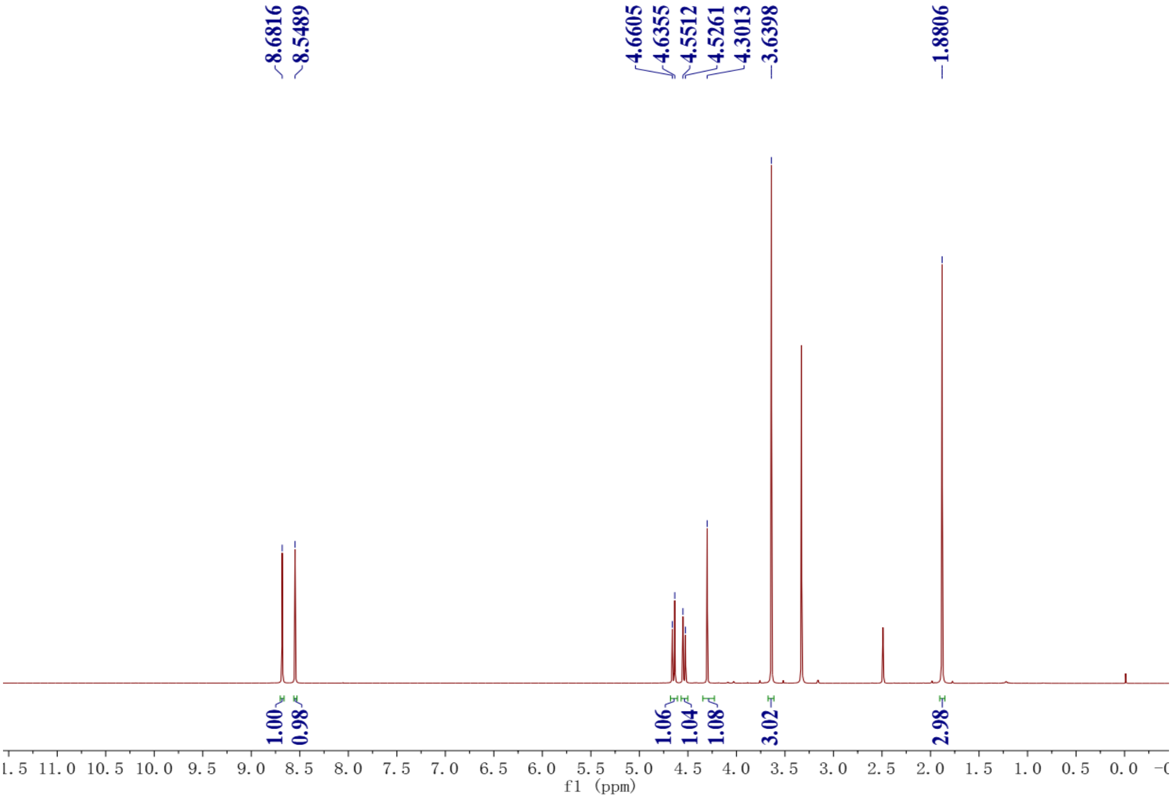

Figure S22. ^{13}C NMR (150 MHz, DMSO-d_6) spectrum of compound **3**

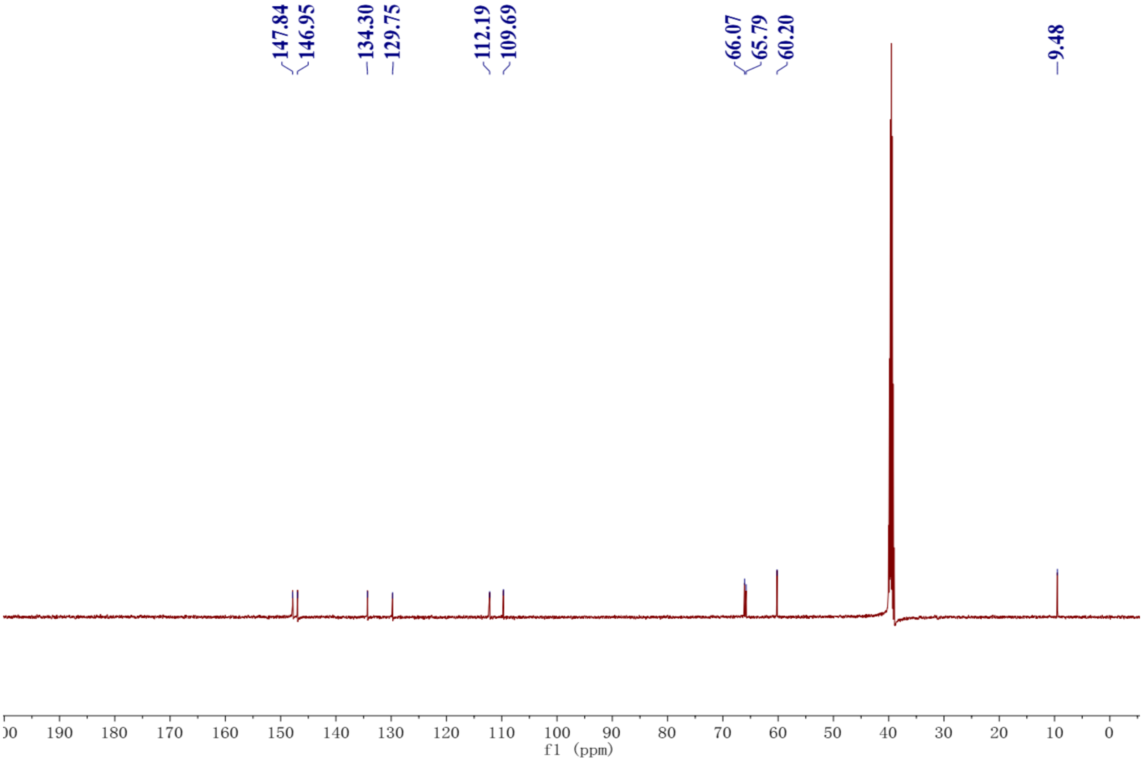

Figure S23. The DEPT spectrum of compound **3** in DMSO-d_6

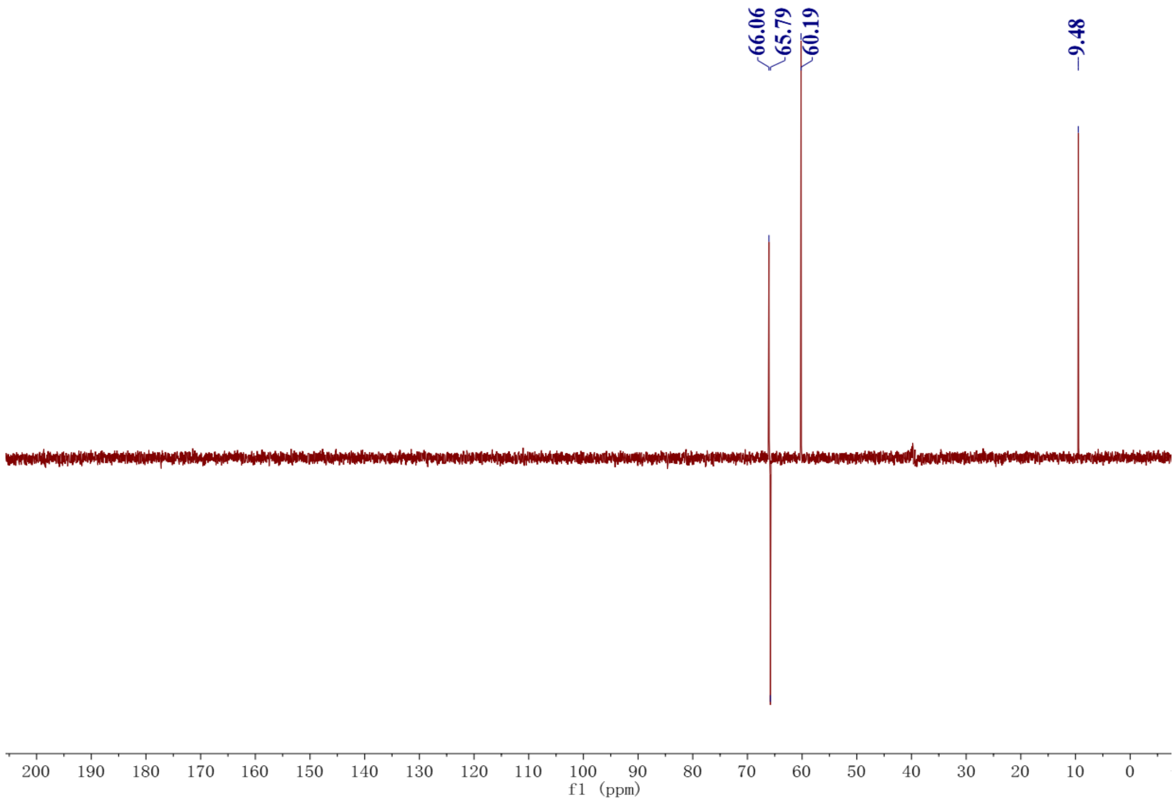

Figure S24. The HSQC spectrum of compound **3** in DMSO-d_6

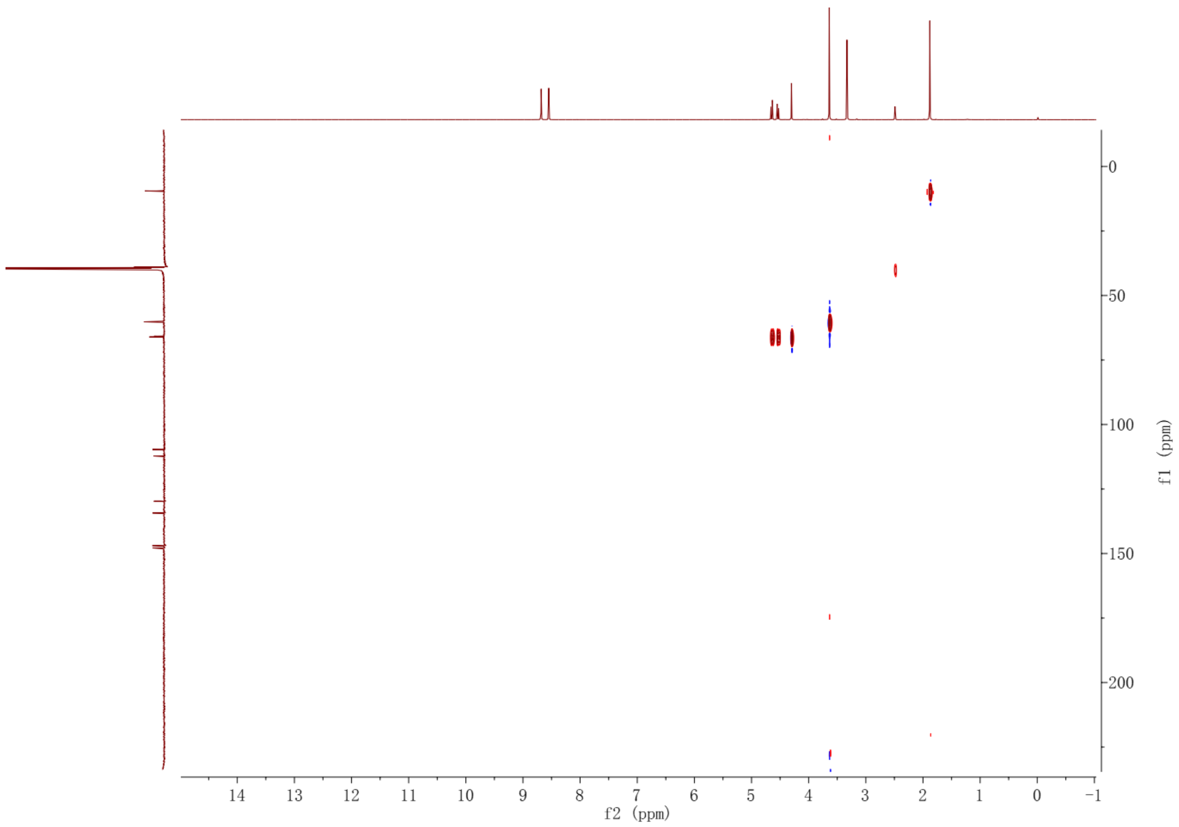

Figure S25. The HMBC spectrum of compound **3** in DMSO-d_6

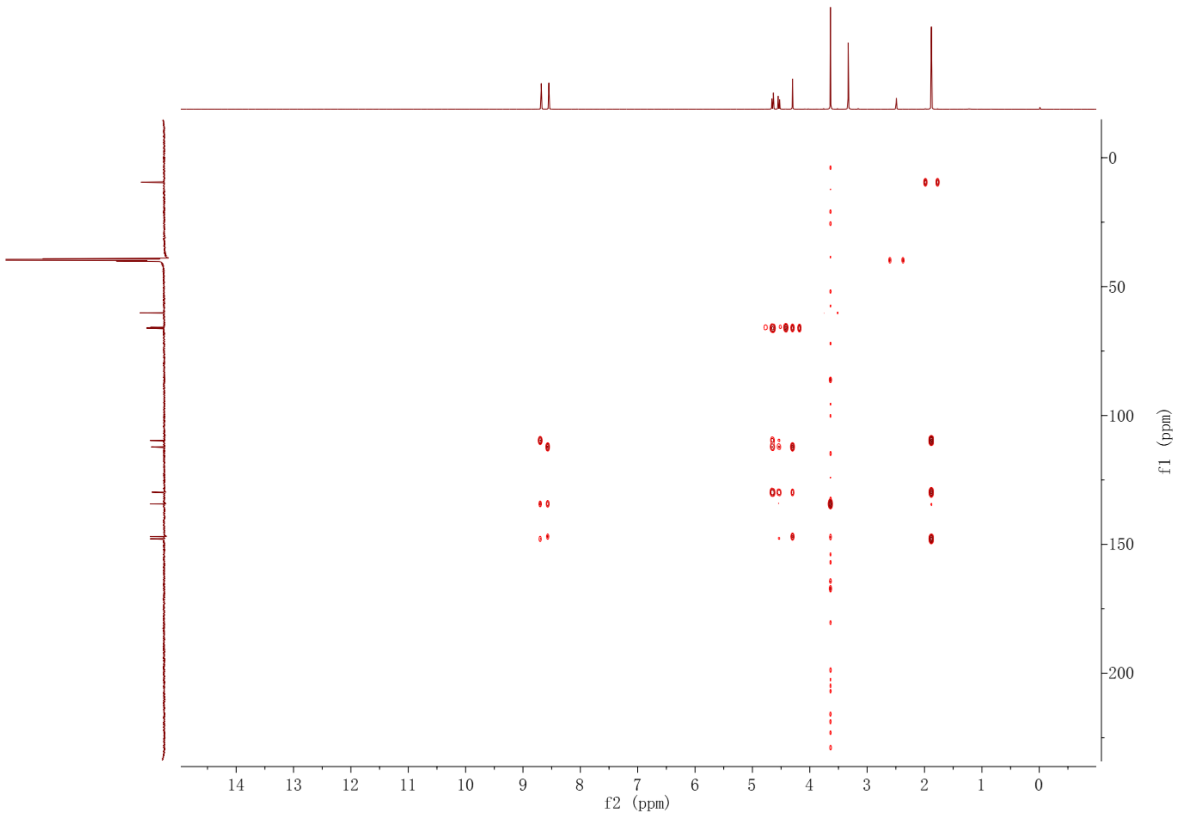

Figure S26. The COSY spectrum of compound **3** in DMSO-d_6

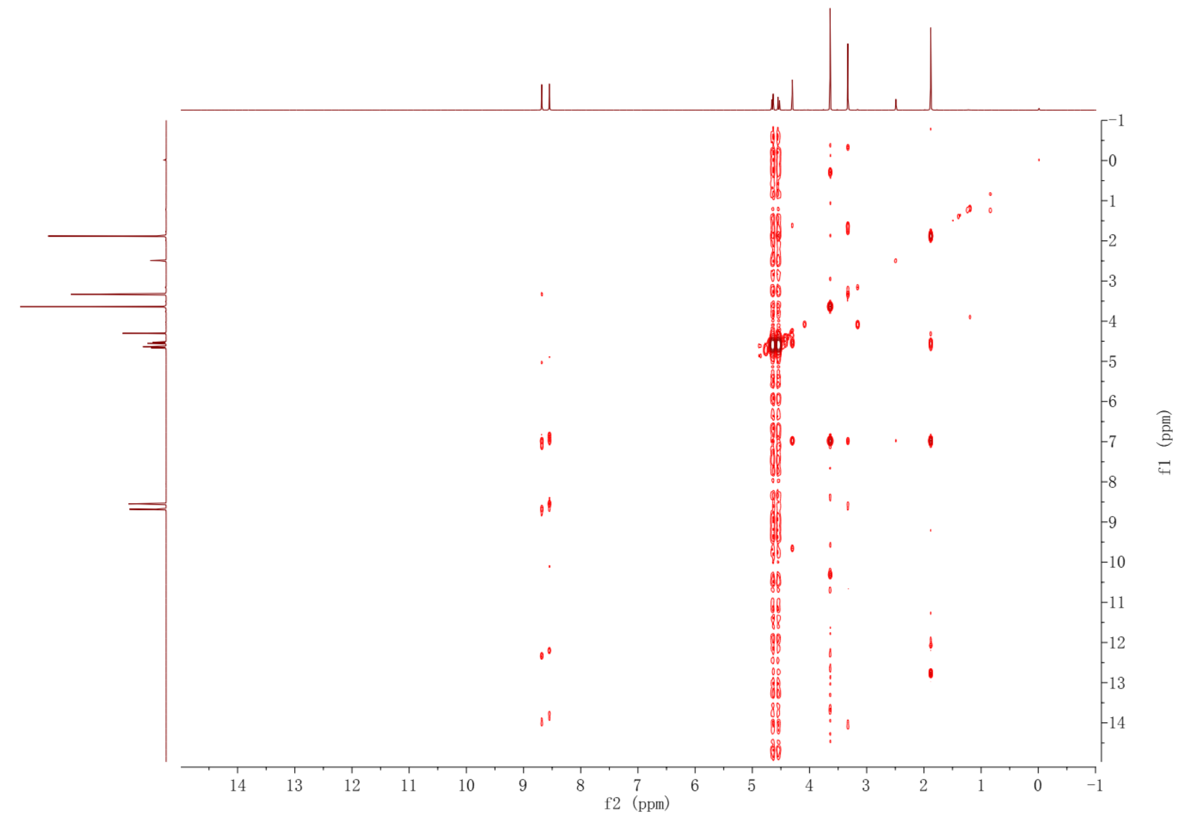

Figure S27. The ROESY spectrum of compound **3** in DMSO-d_6

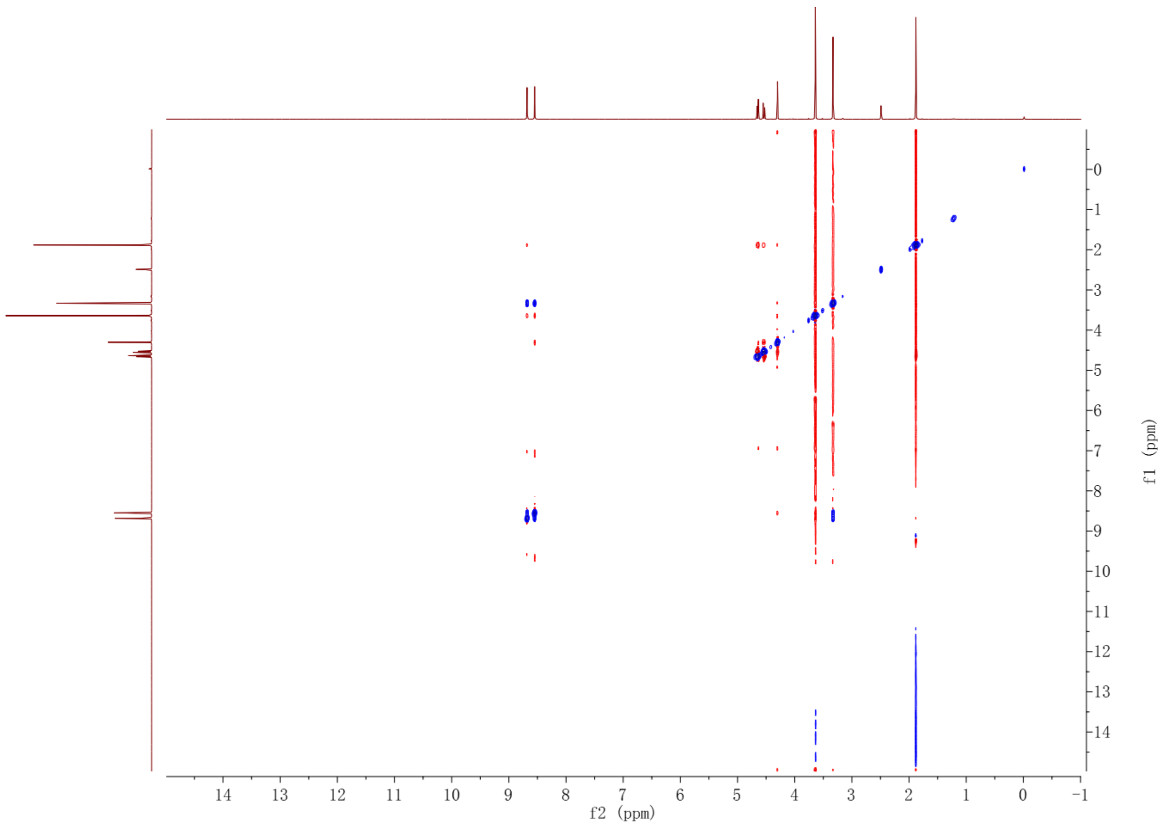

Figure S28. The HRESIMS spectrum of compound **3**

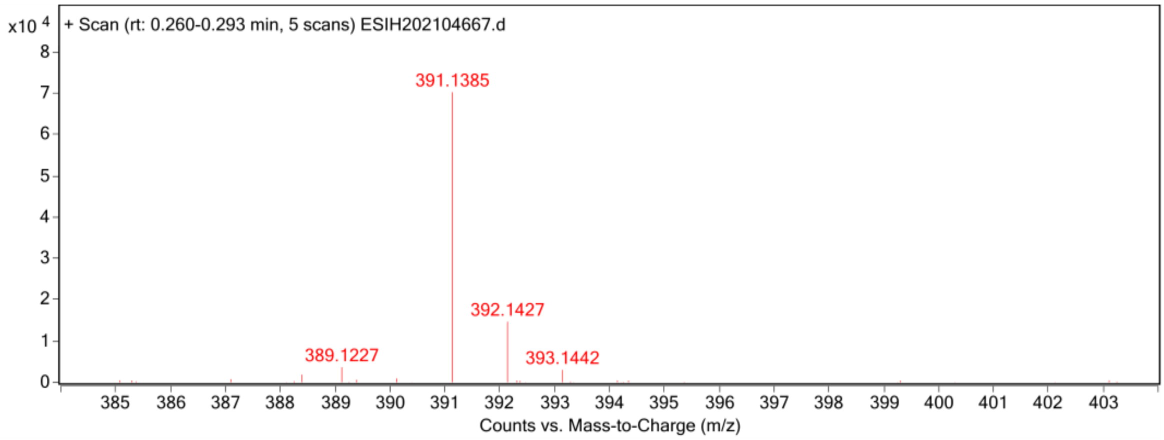

Formula Calculator Results

m/z	Calc m/z	Diff (mDa)	Diff (ppm)	Ion Formula	Ion
391.1385	391.1387	0.29	0.73	C20 H23 O8	(M+H)+

Figure S29. The UV spectrum of compound **3**

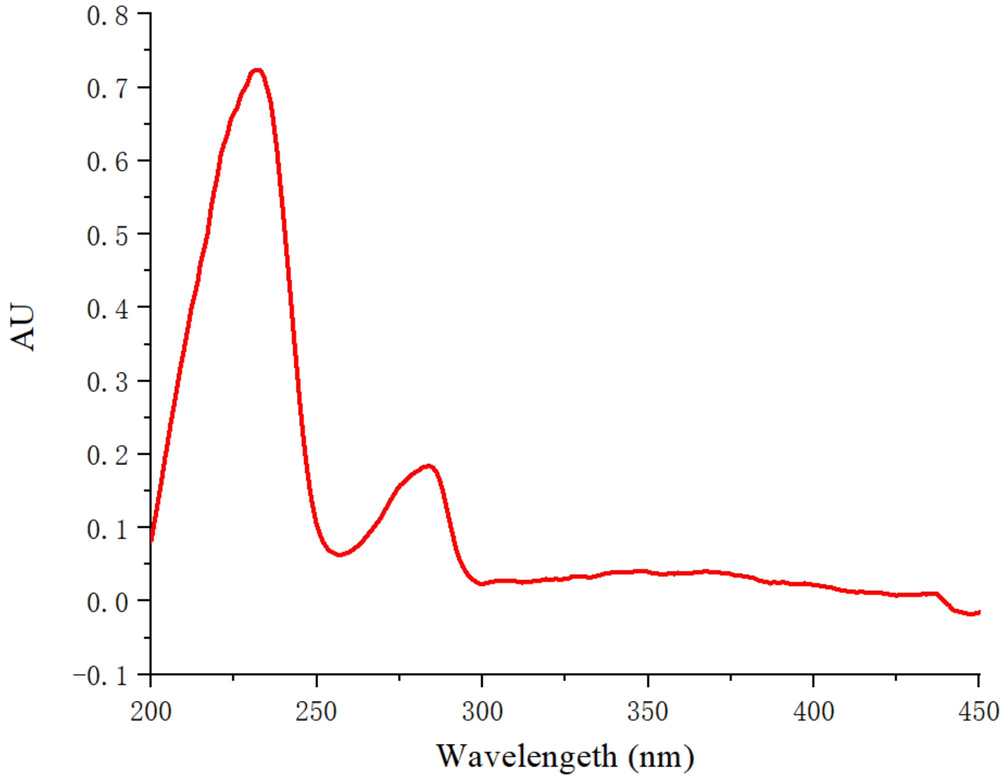

Figure S30. The IR spectrum of compound **3**

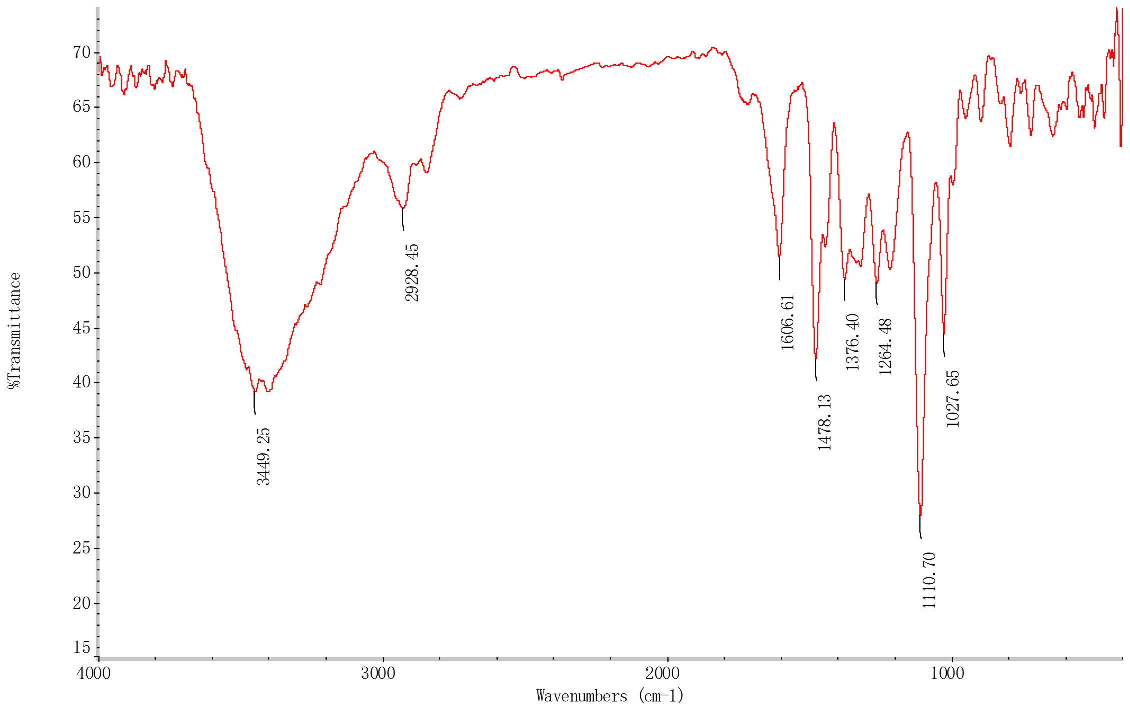

Supplement: Supplementary file 1 [file marinedrugs-21-00157-s001.zip › marinedrugs-2231204-supplementary.pdf]
